# Novel insights into plant defensin ingestion induced metabolic responses in the polyphagous insect pest *Helicoverpa armigera*

**DOI:** 10.1038/s41598-023-29250-3

**Published:** 2023-02-23

**Authors:** Javed A. Mulla, Vaijayanti A. Tamhane

**Affiliations:** Department of Biotechnology (Jointly Merged With Institute of Bioinformatics and Biotechnology (IBB)), Savitribai Phule Pune University, Pune, Maharashtra 411007 India

**Keywords:** Biotechnology, Molecular biology, Environmental sciences

## Abstract

Lepidopteran insect pest *Helicoverpa armigera* is one of the most destructive pests of crop plants and several biotechnological approaches are being developed for its control. Plant defensins are small cationic and cysteine-rich peptides that play a role in plant defense. Ingestion of a defensin from *Capsicum annuum* (CanDef-20) induced a dose-dependent reduction in larval and pupal mass, delayed metamorphosis and also severely reduced fecundity and fertility in *H. armigera*. To understand the molecular mechanisms of CanDef-20 ingestion-mediated antibiosis in *H. armigera* larvae, a comparative transcriptomics analysis was carried out. Predominant downregulation of GOs represents serine-type endopeptidases, structural constituents of ribosomes and integral membrane components and differential upregulation of ATP binding, nucleus and translation, while up-regulation of nucleic acid binding represented by transposable elements, were detected. Different isoforms of lipase, serine endopeptidase, glutathione S-transferase, cadherin, alkaline phosphatase and aminopeptidases were found to be upregulated as a compensatory response to CanDef-20 ingestion. In vitro enzyme assays and qPCR analysis of some representative genes associated with vital cellular processes like metamorphosis, food digestion and gut membrane indicated adaptive differential regulations in CanDef-20 fed *H. armigera* larvae. We conclude that CanDef-20 ingestion affects insect metabolism in a number of ways through its interaction with cell membrane, enzymes, cytoplasmic proteins and triggering transposon mobilization which are linked to growth retardation and adaptive strategies in *H. armigera*.

## Introduction

Insect pests lead to substantial yield losses in crop plants either by direct damage or by the spreading of diseases. Of the number of insect pests that threaten and attack crop plants *Helicoverpa armigera* is polyphagous and the most devastating^1^. Control measures like use of various pesticides and cry transgenic crop-based approaches have been used globally, though *H. armigera* evolves resistance^2^, leading to failure of these methods necessitating development of novel biological approaches for sustainable and environment-friendly pest control.

Polyphagous insects have developed multiple resistance mechanisms like the production of glutathione S-transferase, glucose oxidase, overexpression of insensitive protease and cytochrome P450 mono-oxygenase to cope with plant defenses^3^. The molecular mechanisms of resistance of Bt transgenics have been studied in recent years. The mutation in cadherin and ABCC2 transporter gene present in the brush border epithelium of *H. armigera* and *H. virescens* resulted in the resistance against Bt toxin^4^. In addition to this, altered expression of alkaline phosphatase (ALP)^5^, aminopeptidase N (APN)^6^ and regulatory events in mitogen-activated protein kinase (MAPK)^7^ were the resistance mechanisms used by lepidopteran insects against Bt toxin. These studies provide evidence of notable diversity and plasticity in insect metabolism to counter plant defenses.

Plants have evolved sophisticated regulatory networks to present constitutive and induced defense responses against herbivore attacks^3^. Induction of jasmonate (JA) and salicylic acid (SA)^4^ pathways followed by the production of secondary metabolites, proteinases inhibitor (PIs)^5^, and antimicrobial peptides (AMPs) determine a pest-specific plant defense response. Effect of AMPs on insects is not clearly deciphered, and it is known that there is a much lower probability to generate resistance to AMPs in bacteria than to antibiotics^8^. Therefore, studying the insect’s counter defense mechanism against plant defense molecules like defensin peptides, will help us in pyramiding defense molecules for better insect pest control^9^.

AMPs like defensins were less explored in the context of plant–insect *H. armigera* interactions though their role as antifungal and antibacterial peptides is well studied via their interactions with the cell membranes^10^, cytoplasmic proteins^11^ and transmembrane channels^12^. Plant defensins are cationic peptides with ~ 45–50 amino acids with a conserved three-dimensional structure representing cysteine-stabilized αβ (CSαβ) fold, four to five disulfide bonds and γ-core motif^13^-the major determinant conferring antifungal and antibacterial activity ^14^. Some studies confirm the direct cell membrane binding activity of γ-core motif^15,16^. In our previous work, we have discussed the growth inhibitory potential of *Capsicum annuum* defensin (CanDef-20) on *H. armigera*^17^. CanDef-20 caused around 15% retardation in larval mass and a 12% reduction in pupal mass though the mechanisms through which CanDef-20 induces physiological and morphological implications in the insect pest remained unclear. The present study attempts to decipher the mechanism of action of CanDef-20 in *H. armigera* through a transcriptomics approach.

In this study, we aimed (i) to understand dose-dependent effects of CanDef-20 ingestion in *H. armigera* (ii) elucidate physiological and molecular responses of *H. armigera* fed with recombinant CanDef-20 using a comparative de novo transcriptomics approach and (iii) to understand the correlations in gene expression levels with protein/enzyme activity and adaptive responses of *H. armigera* towards the ingestion of plant defensin CanDef-20. Understanding the role and mechanism of action of plant defensins in insect pests will be a step forward in designing sustainable pest control measures for agriculture.

## Materials and methods

### CanDef-20 amplification and source of insect culture

The *C. annuum* (local variety Phule Jyoti) flower tissues were used for amplification and cloning of CanDef-20 gene as described previously^17^. The *C. annuum* seeds were procured from the local market and grown in a polyhouse. The molecular biology experiments like amplification of the defensin gene, cloning of genes and recombinant expression of proteins was performed after approval from the Institutional Bio-Safety Committee (IBSC-SPPU) as per the guidelines from Department of Biotechnology, Ministry of Science and Technology, Government of India http://dbtbiosafety.nic.in/ or Guidelines and Handbook.

The *H. armigera* eggs were obtained from the National Bureau of Agricultural Important Resources (NBAIR), Bangalore. Eggs were allowed to hatch and neonates were transferred on an artificial diet as described earlier^17^. Only insects with one generation raised in the laboratory were used for bioassays with CanDef-20.

### Cloning and heterologous expression of CanDefensin (CanDef-20) gene

The expression of recombinant CanDef-20 protein and dot blot assay were performed as described previously^17^. For protein expression, the *Pichia pastoris* strain with gene constructs (CanDef-20) or the empty vector (EV) in pPICZ alpha vector were used in buffered methanol complex (BMMY) medium (10 × Yeast Nitrogen Base and buffered with 1 M potassium phosphate buffer pH 6.0) (Hi-media, India). The recombinant proteins were obtained from culture supernatant after 90% ammonium sulphate precipitation and dialysis. Protein samples were subjected to SDS-PAGE using 15% (w/v) polyacrylamide gels^18^. The recombinant proteins expressed using this system carried a C-terminal 6X His and myc tag.

### Dot blot assay of CanDef-20

The recombinant CanDef-20 was confirmed by dot blot assay using rabbit anti-myc primary antibody (Abcam, USA) directed against c-myc tag of the recombinantly expressed CanDef-20. Anti-rabbit secondary antibody was used from Western Blot Development kit (Bangalore genie, India). Finally, the spots were developed by incubating the membrane in BCIP/NBT solution.

### *H. armigera* bioassay

*H. armigera* larval bioassay was performed by using second day neonates maintained on an artificial diet (AD). The AD used in the feeding assay was made up of major and minor components containing ascorbic acid, sorbic acid, yeast extract, cholesterol, vitamins, methyl hydroxy benzoate and chickpea flour which are mixed with agar–agar boiled in water to form a semisolid feed^19^. Total 25 neonates were individually released on AD incorporated with 15.5 and 31.25 μg/ml recombinant CanDef-20 and it was considered as set-1 in a separate small container with diameter of 3 cm. Set-2 with 50 neonates was parallelly run and consists of 62.25, 125, 250 μg/ml recombinant CanDef-20. The AD (control) and EV *P. pastoris* expressed proteins (EV control) were individually run for respective sets. Larval mass, development, mortality, pupal mass, fecundity and fertility was recorded for up to 22 days during the bioassay. All the bioassay sets were maintained at 30 °C with 70% ± 10% humidity with a photoperiod of 16 h. Larval and pupal mass was recorded periodically. Pupae formed were separated in beakers and were monitored for emergence of moths. The moths were fed with 10% sugar solution and allowed to mate in 8:4 and 12:6 (female: male ratio) for set-1 and 2 respectively in a cage (30 × 20 × 15 cm) with a detachable mesh cloth on lid for egg collection. The number of eggs and neonates hatched were recorded.

### RNA isolation and Illumina sequencing

Insect samples (fed with 125 μg/ml recombinant CanDef-20 and EV control incorporated AD) were used for RNA isolation by using TruSeq RNA Sample Prep Kit v2 as per the manufacture’s protocol. Libraries were generated using TruSeq DNA PCR-Free kit following the manufacturer’s recommendations. Sequencing was performed using Illumina HiSeq. 2000 system (Illumina, San Diego, CA, USA) with paired-end reads strategy. The BCL (base calls) binary is converted into FASTQ by using illumina package bcl2fastq (v1.8.4).

### De novo transcriptome assembly and functional annotation of genes

The raw reads were checked using Fastqc tool^20^ and Cutadapt tool^21^ was used for removal of adaptors and low-quality sequences. Also, it was found that sequence files have an average per base Phred score > 30. Normalized reads were assembled into longer fragments (contigs) using Trinity v2.0.6 software^22^. Assembled transcripts were searched for coding transcript by using transdecoder tool^23^. These assembled transcripts were further searched for the orf finding using transdecoder program and the completeness of the transcript. All the protein coding sequences were searched for further annotation using insect uniprot protein database with an e-value cutoff < 1e^−10^. Some differentially expressed uncharacterized genes were further identified by using Uniprot id and BLAST analysis. For further evaluation of the assembly and annotation completeness, BUSCO (Benchmarking Universal Single Copy Orthologs, version 5.4.3) analysis was performed by comparing with arthropod lineage in default settings (http://busco.ezlab.org/).

### Analysis of differentially expressed genes

To compare gene expression profiles across the CanDef-20 and EV control fed *H*. *armigera*, clean reads of each library were mapped to assembly using RSEM software^24^, and abundance estimation was performed in the Trinity package. Expression levels of the transcripts were calculated based on the FPKM normalization method using the edgeR package^25^. Identification of differentially expressed genes (DEGs) was carried out using EV control libraries as a reference. The FPKM values of genes detected in both CanDef-20 fed (treated) and EV control were used to derive ratio (treated/ control), to which a log 2 scale was applied to get fold change values. Transcripts with log_2_FC ≥  ± 2 with *P*-value ≤ 0.05 and FDR ≤ 0.05 were filtered out as the statistically most significant DEGs. To obtain more DEGs and uniquely expressed genes from CanDef-20 fed larvae, transcripts with log_2_FC ≥  + 0.8 and ≤ − 0.8 ratios were also identified. Transcripts with log_2_FC ≥  + 0.8 were considered upregulated in CanDef-20 fed larvae, whereas transcripts with fold change values (**≤ **− 0.8) were considered downregulated. Finally, GO enrichment analysis was performed for DEG sets using manual method and FUNC software^26^.

### Quantitative real-time PCR (RT-qPCR) validation

For RNA extraction, whole larvae pulverized in liquid nitrogen (50 mg) were used from CanDef-20 ingesting 4^th^ instar (125 and 250 μg/ml fed *H. armigera* larvae), EV control and AD fed. These were different insect samples from those used for sequencing but treated under the similar experimental conditions. cDNA was synthesized from 1000 ng of RNA using a verso cDNA Reverse Transcription Kit (Thermofisher, USA) following the manufacturer’s instructions. Primers were designed using NCBI Primer (Supplementary Table S3) and validated by analysis of their PCR amplification efficiencies (E) and correlation coefficients (R2), before the real time analysis. The reaction mixture consists of 1 μl of 0.5-fold diluted cDNA template, 5-μL 2 × Power SYBR® Green PCR Master Mix (Applied Biosystems, Lithuania), 1 μl of each gene-specific primer, and ddH2O to make up the volume. The reaction conditions were as follows: 50 °C for 2 min, 95 °C for 2 min, 40 cycles of 95 °C for 10 s, 60 °C for 30 s; with melt curve 5 s at 95 °C, 65–95 °C, 0.5 °C increase/cycle. The 2^−ΔΔCt^ method was used to detect relative fold change in quantitative real-time PCR and *H. armigera* actin gene was used to normalize the data.

### Enzymatic analysis

The *H. armigera* larvae fed on AD and AD incorporated with 125 μg/ml CanDef-20 and EV proteins respectively were used for detection of total enzyme activities of amylase, protease, lipase, glutathione S-transferase (GST), aminopeptidase and alkaline phosphatase enzymes. Briefly, 50 mg of whole larvae pulverized in liquid nitrogen was homogenized in 200 μl of 0.2 M glycine–NaOH buffer, pH 10.0 and kept at 4 °C for 2 h. The suspension was centrifuged at 4 °C for 20 min at 10,000 rpm and the resulting supernatant was used as *H. armigera* enzyme preparation (HEP). The total protein content of HEP was calculated by Bradford’s method and it was stored at -20 °C till further use.

### Amylase enzyme assay

The amylase activity was assayed by using the 3,5-Dinitrosalicylic acid (DNSA) method^27^. The assay mixture composed of 125 μl of 0.02 M sodium phosphate buffer pH 6.9 containing 6 mM sodium chloride and 40 μg of HEP, which was incubated at 37 °C for 15 min. This enzymatic reaction was terminated with 1.0 ml DNSA reagent, followed by incubation in boiling water bath for 5 min, cooling to room temperature and diluting the reactions 10 times with water and the absorbance measured at 540 nm. The free sugars released at the end of the amylase enzyme activity were estimated using a standard maltose graph.

### Protease enzyme assay

The estimation of the total protease enzyme activity was determined by using Azocasein as a substrate^19,28^ with 40 μg of HEP. Briefly, 40 μg of HEP enzyme was added into 200 µl of 1% azocasein (prepared in 0.2 M glycine–NaOH, pH 10.0) and incubated at 37 °C for 30 min. The reaction was stopped by the addition of 300 µl of 5% trichloroacetic acid. After centrifugation at 12,000 rpm for 10 min, an equal volume of 1 M NaOH was added into the supernatant. The absorbance was measured at 450 nm and one proteinase unit was calculated as the amount of enzyme that increased the absorbance by 1.0 OD under the given assay conditions. The activity of protease was expressed as units/µg/min/ml protein.

### Glutathione S transferase (GST) enzyme assay

The GST activity was estimated by using 1-Chloro-2,4-dinitrobenzene (CDNB) (Sigma, Missouri, USA) as the substrate, following the method described earlier^29^. Reaction mixture (1000 μl) contained phosphate buffer pH = 7 (980 μl), reduced gluthione (GSH) 200 mM (10 μl), CDNB 100 mM (10 μl), and HEP 40 μg. Buffer is added in control reactions instead of the enzyme. Absorption for the reaction mixtures were acquired on Shimadzu UV-1800 spectrophotometer (Cole-Parmer India). The reactions were carried out in 1 ml quartz cuvettes (Shilpent Quartz Cuvette, France) and absorbance at 340 nm was recorded at intervals of 30 s for 15 min at 25 °C. All reactions were done in triplicates and GST specific activity was calculated as μM/ml/min by using 9.6 mM^−1^ as an extinction coefficient for CDNB-GSH conjugate at 340 nm.

### Lipase enzyme assay

Total lipase activity was estimated using the p-Nitrophenyl palmitate (pNPP, Sigma-Aldrich) assay^30^. Solution A consists 0.1 g gum arabica and 0.4 mL Triton X-100 (Hi-media, India) dissolved in 90 ml of distilled water and solution B contained 30 mg pNPP dissolved in 10 ml isopropanol^31^. The complete substrate solution was made by adding 9.5 mL of solution A to 0.5 ml of solution B drop–wise with constant stirring to obtain an emulsion that was stable for two hours. Using this substrate, the lipase activity was determined. 40 μg of HEP was added in the substrate solution and it was incubated at 37^o^C for 30 min. The reaction was stopped by adding 1% sodium carbonate (Na_2_CO_3_) and absorbance of the samples was measured at 410 nm.

### Alkaline phosphatase (ALP) and aminopeptidase (APN) enzyme assays

*P*-nitrophenyl phosphate and leucine-*p*-nitroanilide (Sigma–Aldrich) were used as substrates respectively for detection of specific ALP and APN enzymatic activities as per protocol^32^. 40 μg of HEP were mixed with ALP buffer [0.5 mM MgCl2, 100 mM Tris/HCl (pH 9.5)] consisting 1.25 mM of *p*-nitrophenyl phosphate or APN buffer [0.2 M Tris/HCl (pH 8), 0.25 M NaCl] with 1 mM of leucine-*p*-nitroanilide. ALP enzyme activity was checked as the change in the absorbance at 450 nm for 3 min at 25 °C while APN enzyme activity was measured at 410 nm by checking rate of generation of *p* -nitroaniline.

### Statistical analysis

The Student’s t-test was used for statistical analysis and the level of significance was indicated as *P < 0.05, **P < 0.001 and ***P < 0.0001 by comparing with readings of EV control. The graphs were plotted by using an online demo version of Graphpad prism and Microsoft excel. The model image was created with Biorender.com.

## Results

### Effect of CanDef-20 ingestion on growth and development of *H. armigera*

**Figure 1:**
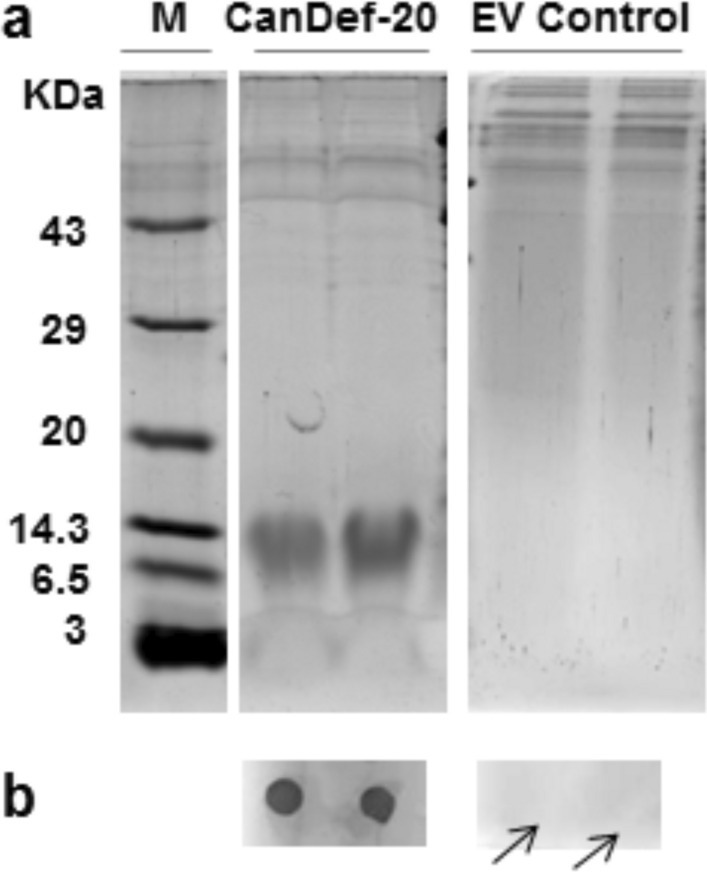
The SDS-PAGE and dot blot analysis of recombinant CanDef-20 and EV proteins: (**a**) CanDef-20, EV protein and protein molecular weight ladder (lane M, Genei Laboratories Private Limited) were resolved on 15% SDS-PAGE and visualized by Coomassie blue staining. (**b**) The rabbit anti-myc primary antibody was used against myc epitope of pPICZ-alpha A vector in dot blot analysis. Secondary antibody provided with Western Blot Development kit (Bangalore genie, India) was used to visualise myc-tagged primary antibody complex.

**Figure 2:**
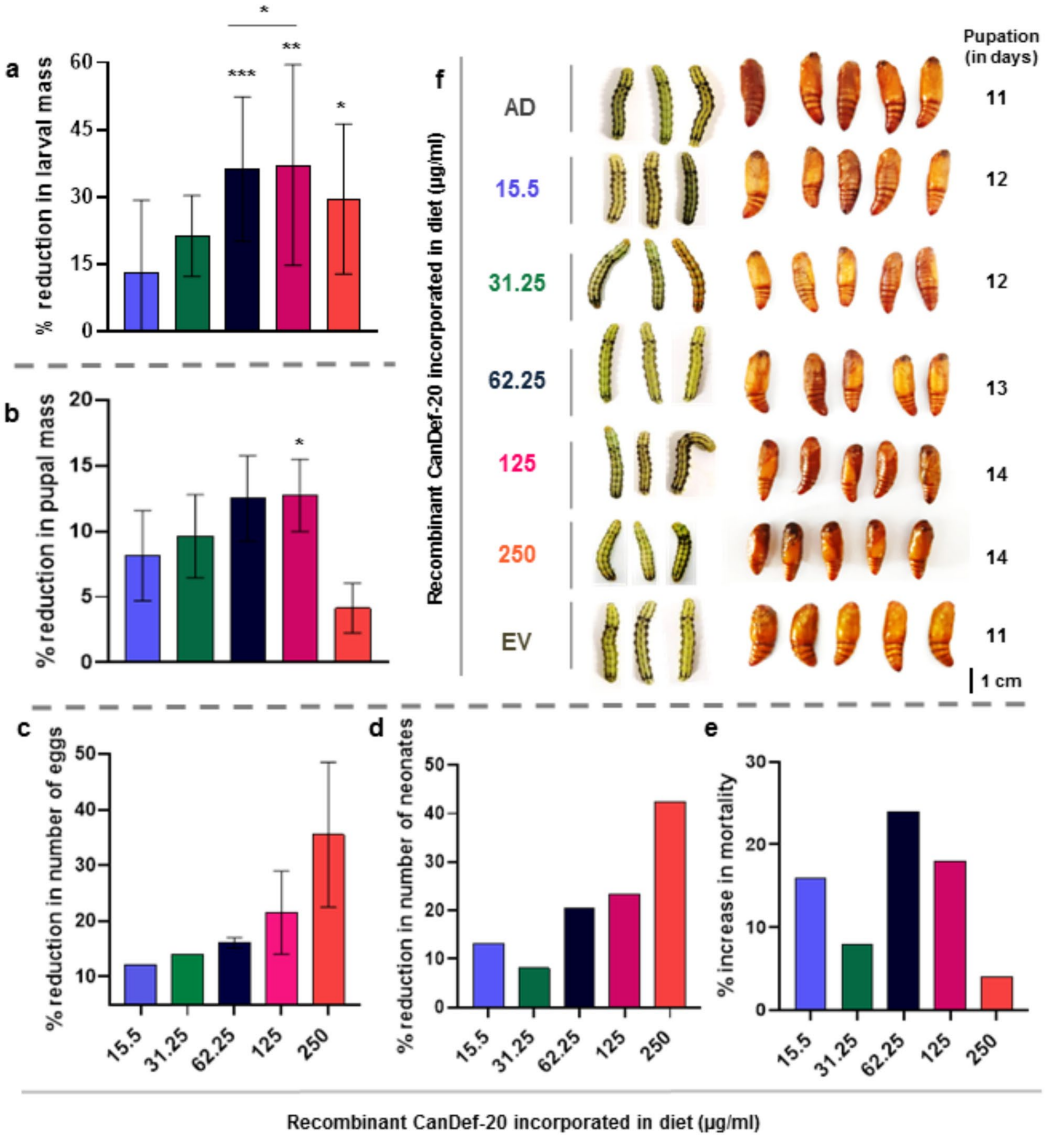
*H. armigera* growth and development in CanDef-20 feeding assay as compared to control (AD) and EV control diet: (**a**, **b**) The % reductions in the larval and pupal mass of *H. armigera* are shown by bar graph respectively. (**c**) The effect of CanDef-20 feeding on fecundity of *H. armigera* larvae is shown by % decrease by respective treatments. (**d**) The number of neonates hatched per 1,000 eggs was recorded for each treatment. (**e**) The mortality induced by different concentrations of CanDef-20 in *H. armigera* larvae. (**f**) The morphological variations in the late fifth instar *H. armigera* larvae and their pupae indicated the pronounced effect of CanDef-20 on larval and pupal growth. The % reduction in larval and pupal mass values are significantly different from EV control at * for *P* < 0.05, ** for *P* < 0.001 and *** for *P* < 0.0001, respectively.

Recombinantly expressed CanDef-20 protein showed a molecular mass of ~ 14 kDa and their dot blot assay with anti-myc antibodies confirmed the presence and expression of the recombinant proteins (Fig. 1a,b). The original images of gels and dot blot are given in Supplementary Fig. S1. The effect of CanDef-20 on the development of *H. armigera* was evaluated by measuring the larval mass, pupal mass, fertility and fecundity in insects fed on various doses of the recombinant protein incorporated in artificial diet (15.5, 31.25, 62.25, 125 and 250 μg/ml). *H. armigera* larvae ingesting recombinant CanDef-20 displayed a dose dependent effect on larval mass as compared to the larvae ingesting control diets (EV and AD fed) (Supplementary Fig. S2). Maximum reduction in the mass of larvae (37%) was observed when the larvae were fed 125 μg/ml of CanDef-20 containing diet (Fig. 2a). Similarly, reduction in pupal mass (4% to 12%) was also observed in the larvae fed with CanDef-20 (Fig. 2b and Supplementary Fig. S2). Interestingly, upon ingestion of 250 μg/ml CanDef-20, *H. armigera* larvae indicated a slight yet significant increase in larval and pupal mass compared to larvae ingesting 125 μg/ml CanDef-20, though the mass was still lower than EV control ingesting larvae. Additionally, around 48–72 h delay in pupation was observed in insects fed with 125 and 250 μg/ml CanDef-20. The egg laying and egg hatching were also affected in *H. armigera* moths raised from larvae fed with higher concentrations of CanDef-20. 12–21% reduction was observed in total number of eggs laid by *H. armigera* moths raised from CanDef-20 fed larvae (Fig. 2c). The egg fertility was seen to be reduced from 13 to 68%. 250 μg/ml CanDef-20 feeding resulted in lowest number (440) of neonates emerging per 1,000 eggs (Fig. 2d). Larvae feeding on 62.25 μg/ml CanDef-20 diets showed high mortality (24%) amongst all tested concentrations (Fig. 2e). CanDef-20 ingestion induced larval and pupal mass reduction was also evident from the comparative morphologies of late fifth instar *H. armigera* larvae and their pupae (Fig. 2f). The 24–72 h of delayed pupation was observed in the larvae fed with CanDef-20. The day where ~ 70% larvae entered to pupation was showed in Fig. 2f. The delayed metamorphosis effects of CanDef-20 on the larvae and pupae of *H. armigera* are also evident in a separate larval bioassay with 150 μg/ml and 300 μg/ml CanDef-20 (Supplementary Fig. S2).

### Comparative transcriptome analysis of *H. armigera* larvae fed with CanDef-20 and EV incorporated diets

The feeding experiments demonstrated a dose-dependent reduction in larval and pupal mass of *H. armigera* when ingested up to 125 μg/ml CanDef-20 containing diet. The 4^th^ instar *H. armigera* larvae fed for 9 days on 125 μg/ml CanDef-20 or fed on EV protein containing diet were used for a comparative transcriptomic analysis using RNA-seq.

Illumina sequencing of *H. armigera* libraries yielded 1091.8 and 1278.1 million read pairs for CanDef-20 and EV diet fed larvae respectively. The de novo assembly analysis of clean reads resulted in a total of 1,57,768 transcripts of which 65,535 assembled transcripts (41%) were > 200 bp in length and were annotated with UniProtKB insect data resulting in13,779 transcript annotations (Table 1). Out of the 13,779 annotated transcripts 6982 showed > 75% query coverage and these were used for further comparative transcriptome analysis of DEGs between CanDef-20 and EV fed *H. armigera* larvae. Of the 6982 genes, 47% were found to align with genus *Heliothis* and 6.19% aligned with genus *Helicoverpa*. Applying the criteria of log_2_FC ≥  ± 2 with *P*-value ≤ 0.05 and FDR ≤ 0.05 to the 13,779 transcripts resulted in detection of 2012 transcripts of which 56 transcripts were found upregulated and 529 were downregulated.

**Table 1:** Transcriptomic assembly statistics of *H. armigera* larvae fed with CanDef-20.

Samples		Raw reads		Clean reads	
CanDef-20 Fed		10,918,133		10,593,388	
EV Control Fed		12,781,063		12,436,430	
Total assembled transcripts			157,768		
Number of Unigenes			142,916		
Unigenes (> 200 nucleotide)			65,535		
Transcripts annotated (with insect NR database)			13,779		
Transcripts with best query coverage ≥ 75%			6982		
log_2_FC ≥ ± 2with *P*-value ≤ 0.05 and FDR ≤ 0.05 (2012 transcripts)					
Upregulated transcripts			56		
Downregulated transcripts			529		
log_2_FC ≥ + 0.8 ratio and ≤ -0.8)ratio (treated to control) (6982 transcripts)					
Upregulated transcripts			659		
Downregulated transcripts			2327		
Unique in CanDef-20 fed (treated)			1679		
Unique in EV control fed (only found in control)			1545		
Transcript (*P* value** ≤ **0.05)			1921		
Statistics related to Transcript			Statistics related to Gene		
Contig	N10:	864	Contig	N10:	690
Contig	N20:	559	Contig	N20:	483
Contig	N30:	437	Contig	N30:	397
Contig	N40:	370	Contig	N40:	344
Contig	N50:	324	Contig	N50:	308
Median (contig length)	275		Median (contig length)	271	
Average	337.4		Average	320.28	
Total assembled bases	53,230,840		Total assembled bases	45,772,751	

Applying the criteria of log_2_FC ≥  + 0.8 and ≤ − 0.8 ratios to 6982 transcripts with > 75% query coverage, 2327 (33.32%) genes were found to be downregulated and 659 (9.4%) genes were found to be upregulated upon CanDef-20 feeding. Additionally 1679 genes were found to be uniquely expressed in CanDef-20 fed larvae and 1545 genes were uniquely expressed in EV control fed larvae.

The BUSCO analysis was applied on all assembled transcripts and it detected 19% complete (154 Complete and single-copy BUSCOs, 39 Complete and duplicated BUSCOs), while 24% of as fragmented (243 BUSCOs) and 57% are missing (577 BUSCOs) out of 1013 single copy orthologs for arthropods (Supplementary Table S4).

### Differential gene expression analysis in CanDef-20 fed *H. armigera*

**Figure 3:**
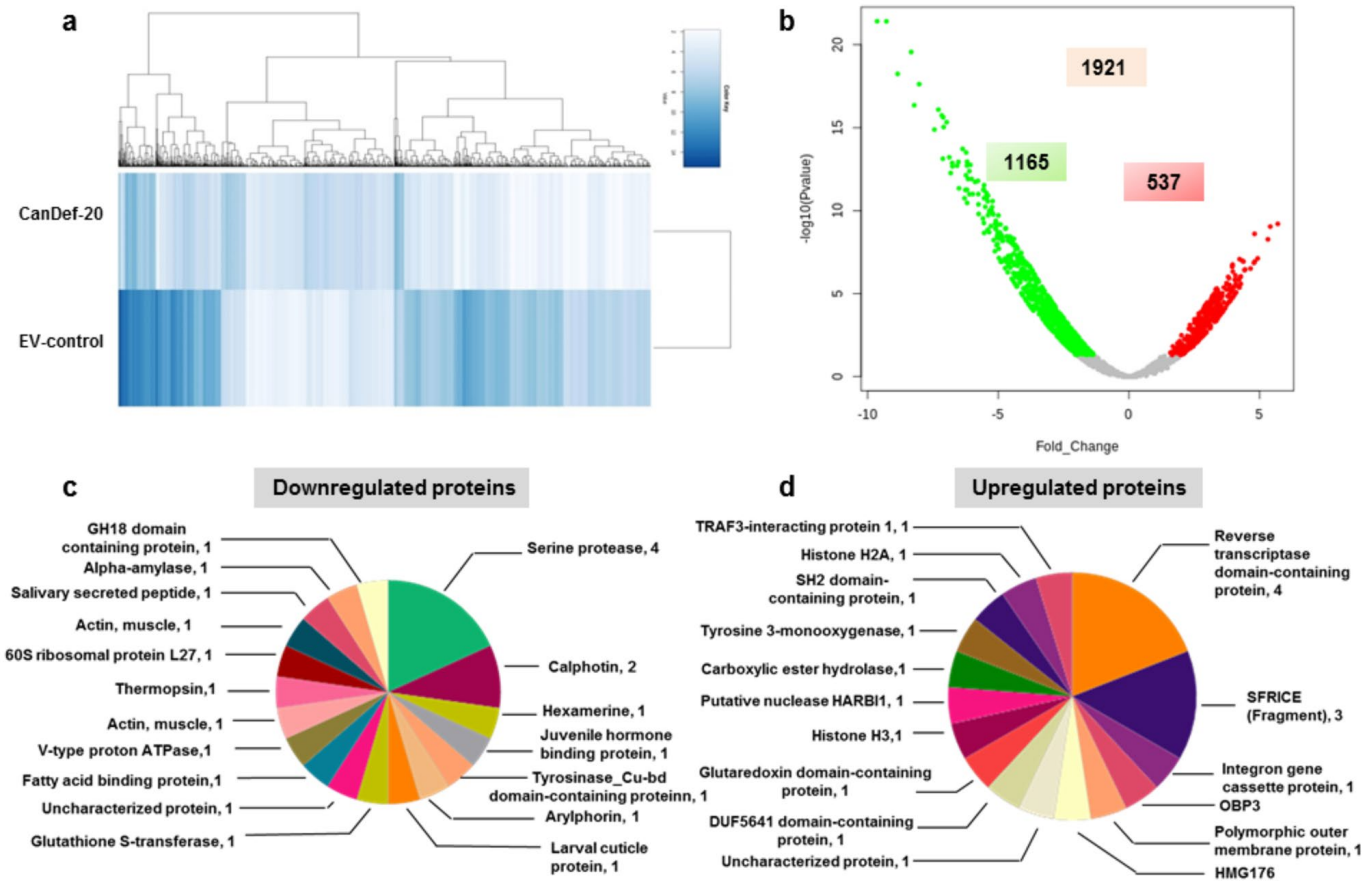
Transcriptomic analysis: (**a**) Hierarchical clustering analysis of differentially expressed genes between CanDef-20 fed and EV control fed *H. armigera* larvae. (**b**) Volcano plot is showing statistically significant gene expression*.* The down and up regulation was denoted by green and red dots respectively. (**c**) and (**d**) Top 25 down and upregulated DEG’s are shown by pie chart respectively. The number of transcripts appeared for particular gene is denoted.

The overall gene expression pattern in the control and CanDef-20 fed larvae was determined by hierarchical clustering and results indicated a prominent downregulation of most genes in CanDef-20 fed *H. armigera* (Fig. 3a). The volcano plot indicated significant differential regulation of 1702 out of 1921 transcripts with *P*-value ≤ 0.05 (Fig. 3b; Table 2). The analysis with ≥ two fold criteria with *P*-value ≥ 0.05 and FDR ≥ 0.05 showed juvenile-hormone binding protein (JHBP), hexamerin, serine protease, arylphorin and calphotin transcripts represented amongst the highly downregulated transcripts while reverse transcriptase domain containing protein, putative nuclease HARBI1, carboxylic ester hydrolase and tyrosine 3-monooxygenase were represented amongst the highly upregulated transcripts (Fig. 3c and 3d; Supplementary Fig. S3; Supplementary Table S1). Also, juvenile hormone-suppressible protein, serine protease and some isoforms of glutathione S-transferase were uniquely found in EV control larvae with very high FPKM values ranging from 6054–1386 whereas some isoforms of arginine kinase, endonuclease-reverse transcriptase and serine protease were uniquely detected in CanDef-20 fed larvae and had high FPKM values ranging from 211–83. A single transcript of GST was upregulated (TRINITY_DN83033_c9_g1_i1 with FPKM 2.44) and other isoform of GST (TRINITY_DN82857_c2_g2_i2 with FPKM 8.44) was also found amongst genes uniquely expressed in CanDef-20 fed *H. armigera* larvae.

**Table 2:** Top 10 upregulated and downregulated proteins in CanDef-20 fed insect shown in volcano plot.

Sr. No	Transcript id	Fold change	-(log P value)	Matched uniprot id	Protein names	Organism
**Upregulated proteins**						
1	TRINITY_DN83412_c6_g2_i1	5.694941	9.201742	A0A2H1WG43	SFRICE_037529	*S. frugiperda* (Fall armyworm)
2	TRINITY_DN82568_c3_g1_i3	5.408217	9.038418	P18488	Homeotic protein empty spiracles	*D. melanogaster* (Fruit fly)
3	TRINITY_DN83065_c3_g5_i1	5.318082	8.268862	A0A2R7VU22	Uncharacterized protein	*O. fasciatus* (Large milkweed bug)
4	TRINITY_DN82238_c11_g4_i1	4.925622	7.120954	A0A1Y1L125	Uncharacterized protein Nucleoporin NUP188 homolog	*P. pyralis* (Eastern firefly)
5	TRINITY_DN82995_c2_g1_i1	4.815952	6.935561	A0A1W4WR17	Twitchin	*A. planipennis* (Emerald ash borer)
6	TRINITY_DN82945_c1_g1_i1	4.806776	8.599529	A0A151J635	Uncharacterized protein CCHC-type domain-containing protein	*T. cornetzi*
7	TRINITY_DN82860_c12_g1_i1	4.781217	6.848041	A0A2A4JYZ1	Uncharacterized protein Reverse transcriptase domain-containing protein	*H. virescens* (Tobacco budworm moth)
8	TRINITY_DN83005_c2_g1_i8	4.652604	6.511229	A0A2H1VQ04	SFRICE_039558	*S. frugiperda* (Fall armyworm)
9	TRINITY_DN82539_c5_g2_i2	4.444028	6.456275	A0A2A4IVF1	Uncharacterized protein Superoxide dismutase	*H. virescens* (Tobacco budworm moth)
10	TRINITY_DN81078_c1_g1_i2	4.426557	6.427306	A0A182IYI1	Uncharacterized protein	*A. atroparvus*
**Downregulated proteins**						
1	TRINITY_DN83198_c0_g1_i2	− 9.6387	21.40195	A0A2A4JMW0	Uncharacterized protein-basic juvenile hormone-suppressible protein 1	*H. virescens* (Tobacco budworm moth)
2	TRINITY_DN82746_c0_g1_i1	− 9.2829	21.3964	Q68YP2	Hexamerine	*H. armigera* (Cotton bollworm)
3	TRINITY_DN83202_c0_g3_i3	− 8.8531	18.23386	A0A0K8SDP7	Uncharacterized protein-Calphotin	*L. hesperus* (Western plant bug)
4	TRINITY_DN83234_c0_g1_i6	− 8.3346	19.55163	A0A2A4J4S9	Uncharacterized protein arylphorin subunit beta-like	*H. virescens* (Tobacco budworm moth)
5	TRINITY_DN83234_c0_g1_i2	− 8.2174	16.34541	G3LF43	Arylphorin	*H. armigera* (Cotton bollworm)
6	TRINITY_DN82063_c0_g1_i6	− 8.02709	17.61496	A0A0K8T7D1	Uncharacterized protein Actin cytoskeleton-regulatory complex protein PAN1-like	*L. hesperus* (Western plant bug)
7	TRINITY_DN83386_c1_g3_i11	− 7.4470	14.87827	O18447	Serine protease (Trypsin-like protease)	*H. armigera* (Cotton bollworm)
8	TRINITY_DN82805_c0_g1_i4	− 7.2939	16.0792	O18439	Diverged serine protease	*H. armigera* (Cotton bollworm)
9	TRINITY_DN82625_c1_g1_i1	− 7.1640	15.72952	O02443	Larval cuticle protein 1	*H. armigera* (Cotton bollworm)
10	TRINITY_DN83068_c1_g1_i1	− 7.1219	13.12375	A0A2A4J8G1	Uncharacterized protein chitinase-like protein EN03 isoform X4	*H. virescens* (Tobacco budworm moth)

**Figure 4:**
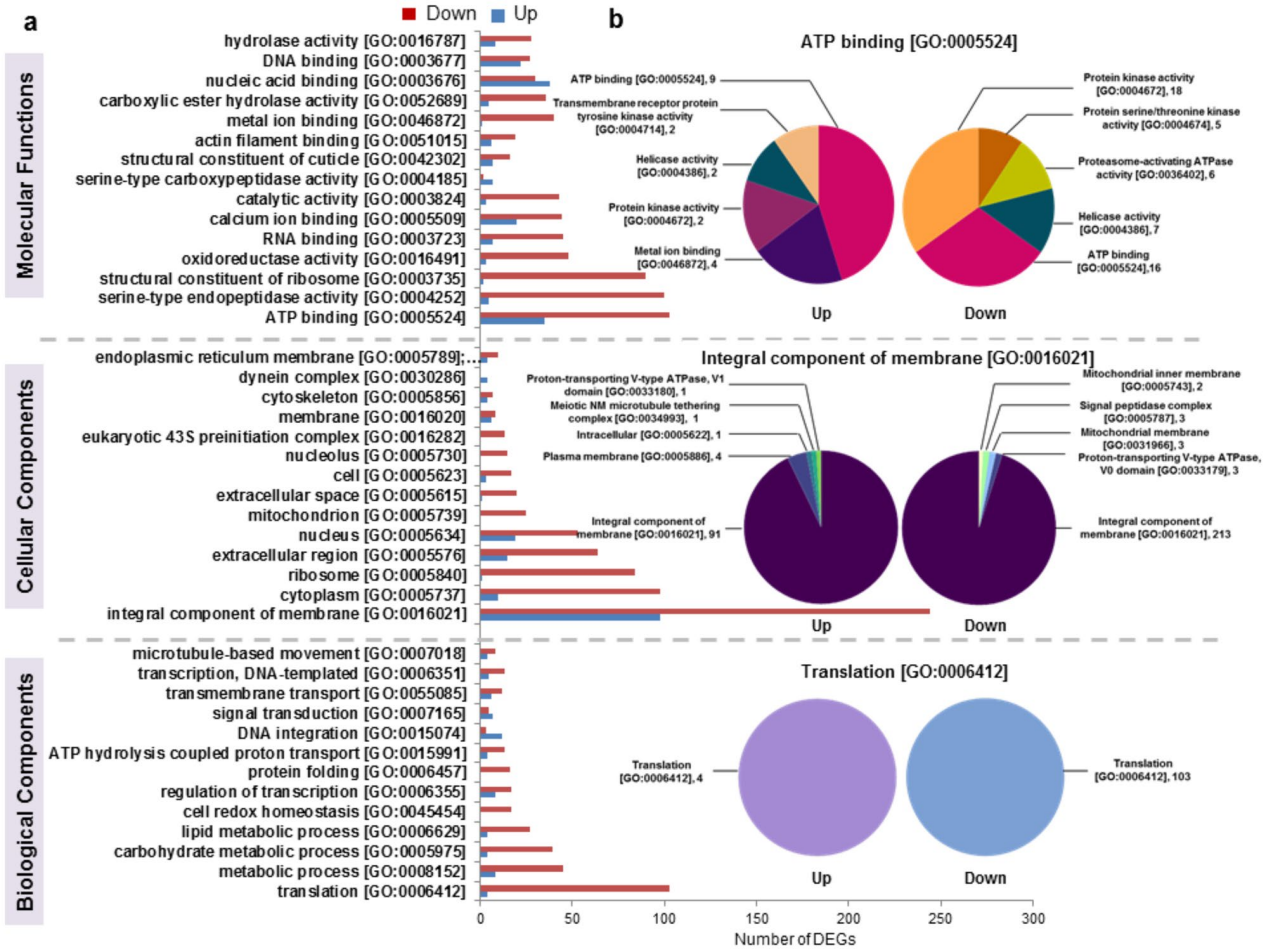
Gene Ontology enrichment of DEGs of CanDef-20 fed insects compared to EV control: (**a**) Gene Ontology (GO) classifications of transcripts into three categories like molecular function, cellular component and biological process. The X-axis corresponds to the number of DEGs appeared in analysis while the Y-axis represents different GOs. Red and blue bars are showing down and up regulation respectively. (**b**) The sub categorization of top most GO is further analyzed and shown in the form of pie chart. The name and occurrence of sub GO is denoted.

**Figure 5:**
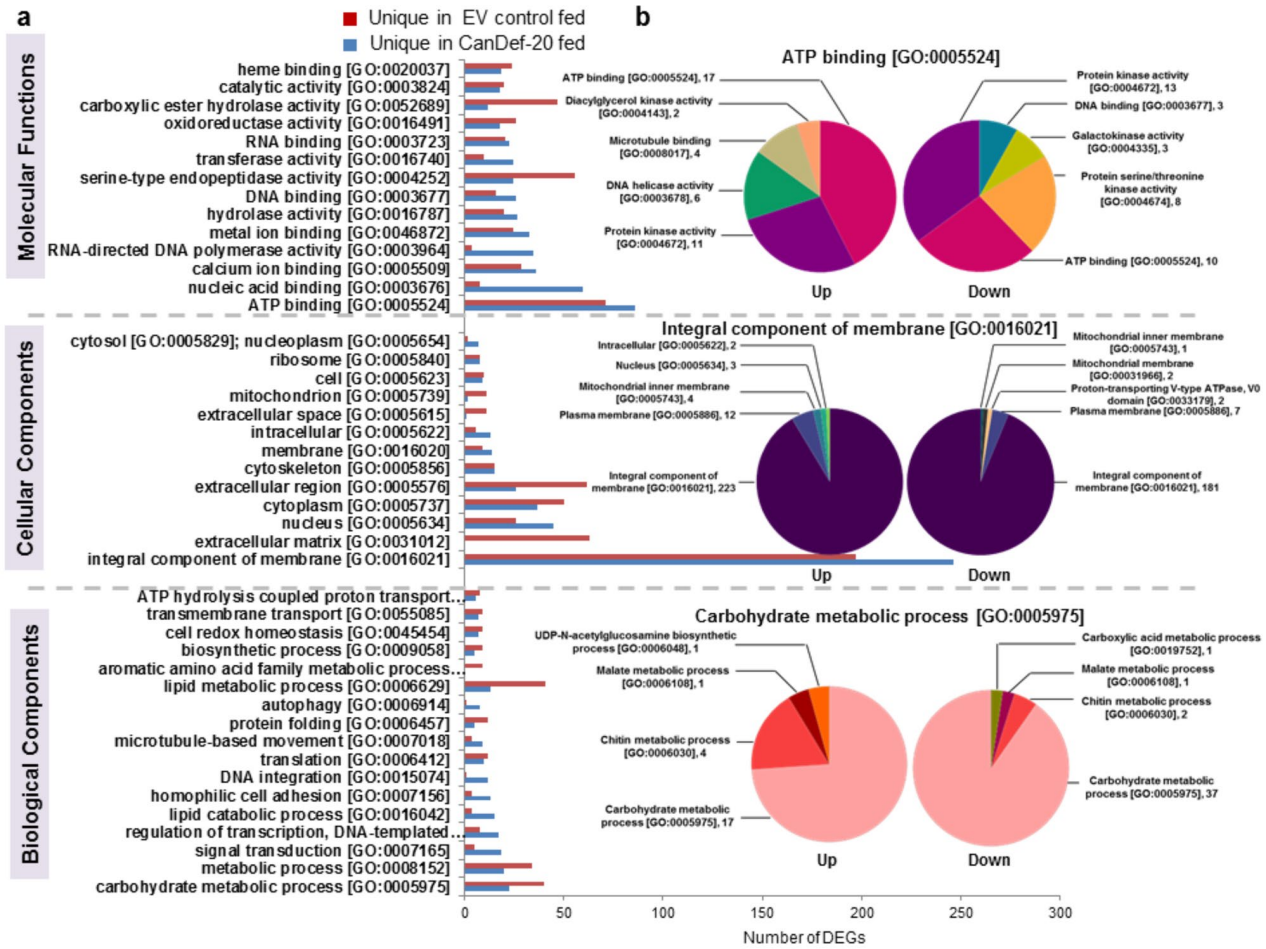
Gene Ontology enrichment of DEGs of uniquely expressed in CanDef-20 and EV fed insects: (**a**) Gene Ontology (GO) classifications of transcripts into three categories like molecular function, cellular component and biological process. The X-axis corresponds to the number of DEGs appeared in analysis while the Y-axis represents different GOs. Red and blue bars were showing unique expression in EV control and CanDef-20 fed respectively. (**b**) The sub categorization of top most GO is further analyzed and shown in the form of pie chart. The name and occurrence of sub GO is denoted.

A comparative GO enrichment for differentially expressed genes with log_2_FC ≥  + 0.8 and ≤ − 0.8 ratios criteria was carried out and top10 GOs are represented (Figs. 4 and 5). Majority of top10 GOs from the differentially expressed set were represented by downregulated genes in *H. armigera* upon CanDef-20 ingestion (except the GO nucleic acid binding, which was upregulated) (Fig. 4); while the uniquely expressed set was represented by majority of genes induced by CanDef-20 (Fig. 5). Integral component of membrane, ATP binding and translation were found to be most prominent differentially regulated GOs in the CanDef-20 fed larvae (Fig. 4a). The subcategories of top most differentially regulated GOs are represented in Fig. 4b. GO ATP binding subcategorized into ATP dependent enzymatic processes like protein kinase, proteasome activating ATPase and helicase; while the GO integral component of membrane included sub GOs like mitochondrial membrane associated genes, signal peptidase complex, proton transporting V-type ATPase, plasma membrane and microtubule tethering complex. Uniquely expressed GOs showed prominence of integral component of membrane, ATP binding and carbohydrate metabolic process (Fig. 5a and b).

**Figure 6:**
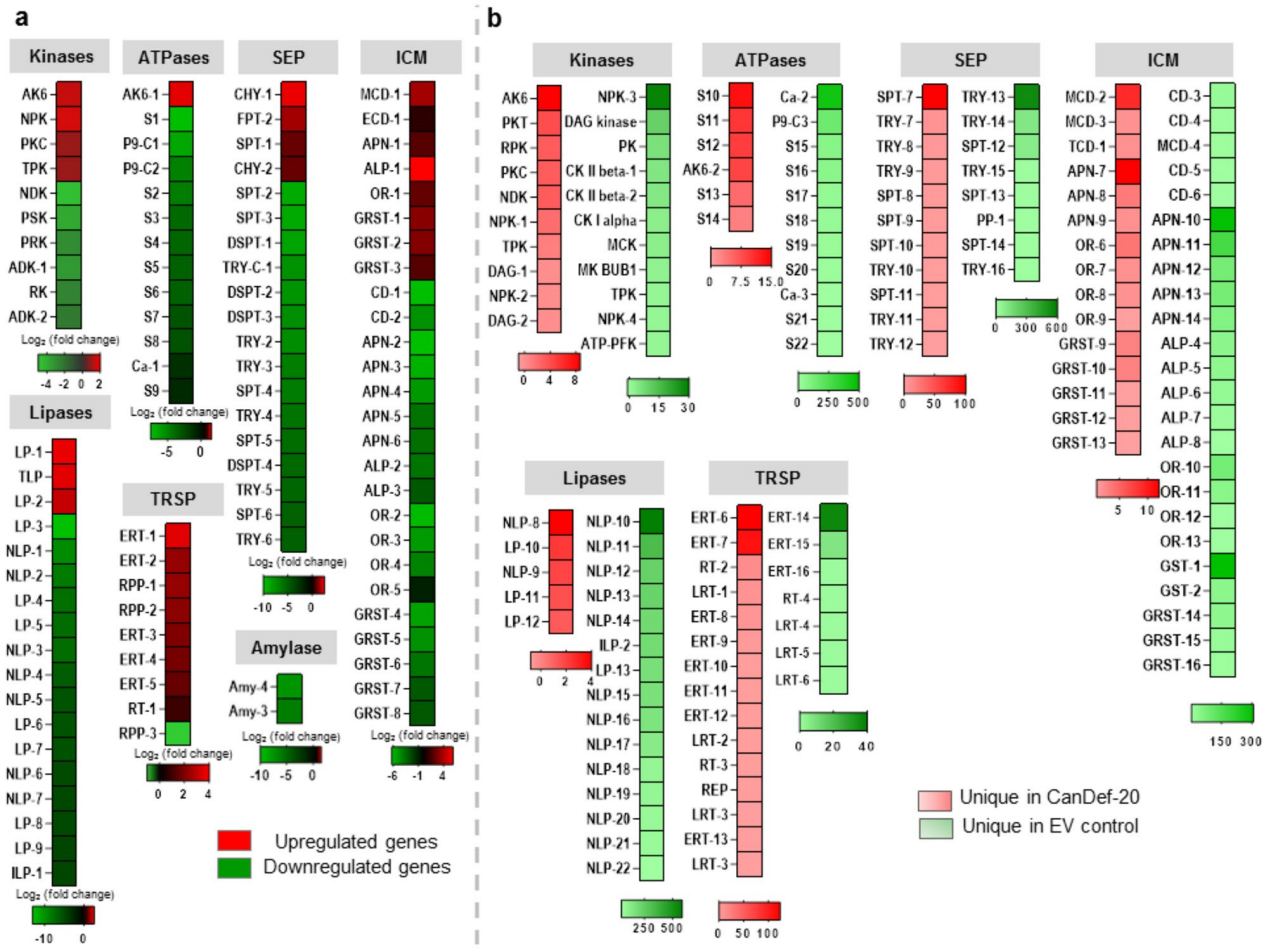
Differential and unique expression pattern of some selected gene categories: (**a**) CanDef-20 induced differential regulation of *H. armigera* genes from selected GOs is represented by the heatmaps. Different gene isoforms of kinases, ATPases, serine-endopeptidases (SEP), integral membrane components (ICM), lipases and transposons (TRSP) are shown on the basis of log_2_ fold change. The down and up regulation is shown by green and red colored gradient respectively. (**b**) The unique expression pattern of different gene isoforms of kinases, ATPases, serine-endopeptidases (SEP), integral membrane components (ICM), lipases and transposons (TRSP) are detected in CanDef-20 and EV control fed larvae separately and represented by the heatmaps. The unique expression pattern in CanDef-20 fed larvae is shown by red gradient. The green gradient is used to show unique expression in EV fed larvae.

Some prominent GOs namely kinases, ATPases, serine-endopeptidases, integral membrane components, lipases and transposons—appeared in differentially expressed as well as uniquely expressed sets. Different isoforms of the above mentioned genes were detected and found to have variable expression levels as represented by the heatmaps (Fig. 6a and b; Supplementary Table S2). Downregulated isoforms of genes were balanced by few upregulated isoforms indicating a CanDef-20 induced compensatory response in *H. armigera*. For example, in GO transposable elements, isoforms were found to be highly upregulated, while in GO lipase interestingly only three isoforms were found to be upregulated (LP1, TLP and LP2) and > 15 downregulated. LP1 and TLP are not characterized from *H. armigera* (Supplementary Table S2), though they showed homology with lipase-1 (NM_001043501.1) and triacylglycerol lipase (XM_038014482.1) respectively found in *Bombyx mori*. The third (LP2) upregulated lipase has homology with *H. armigera* triacyl glycerol lipases (XM_047184139.1).

### CanDef-20 fed *H. armigera* larvae have higher activities for selected enzymes

**Figure 7:**
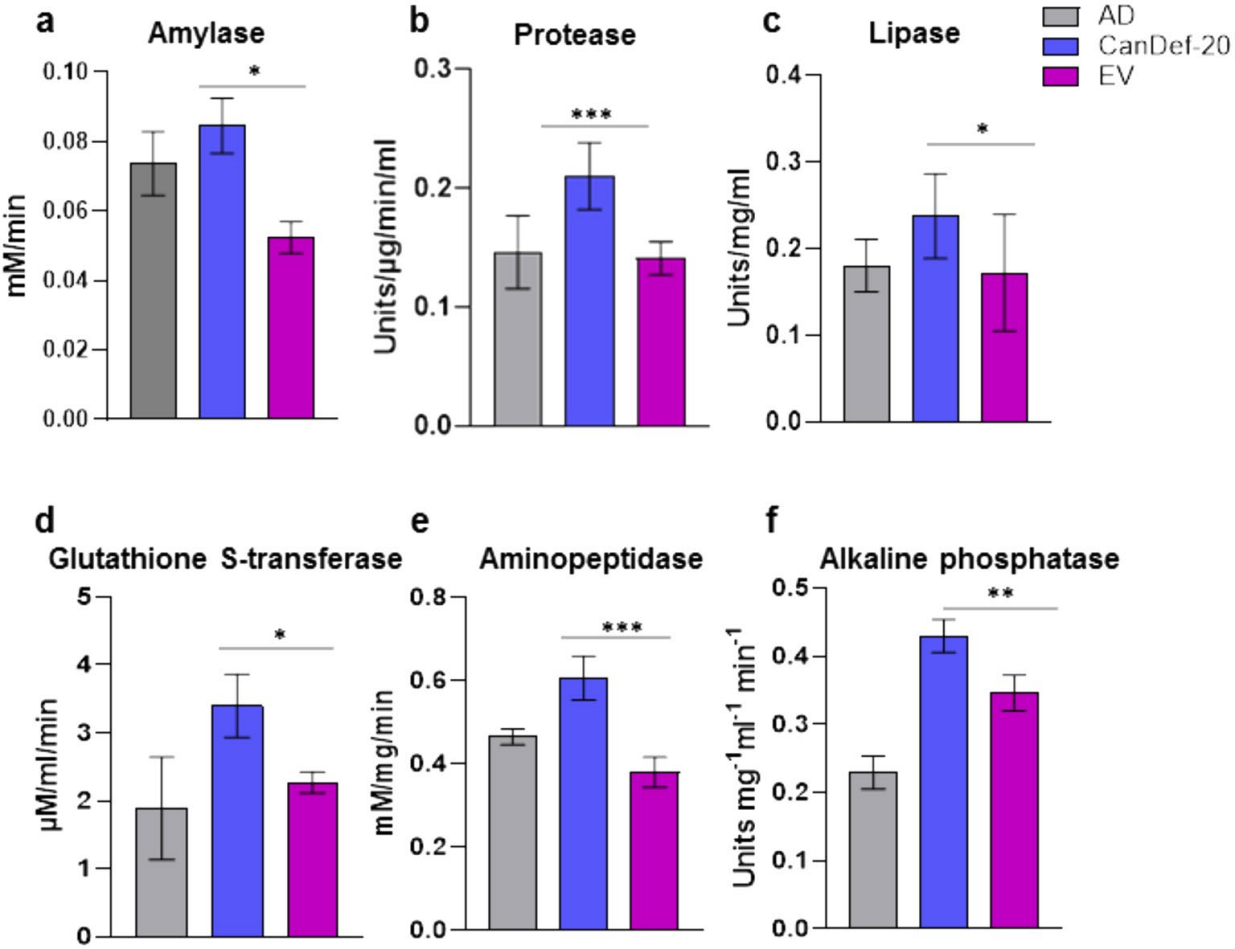
Effect of CanDef-20 feeding on the enzyme activities: The *H. armigera* enzyme preparation (HEP) was used in the assays to detect (**a**) amylase, (**b**) protease, (**c**) lipase, (**d**) glutathione S-transferase (GST), (**e**) aminopeptidase and (**f**) alkaline phosphatase enzyme activities and is shown by bar graph. The enzyme activities from artificial diet (AD), recombinant CanDef-20 incorporated diet (CanDef-20) and empty vector expressed proteins (EV) were analyzed. The total enzyme activity values are significantly different from EV control at * for *P* < 0.05, ** for *P* < 0.001 and *** for *P* < 0.0001 respectively.

HEP from CanDef-20 fed and EV control protein fed *H. armigera* was analyzed for detection of amylase, protease, lipase, GST, aminopeptidase and alkaline phosphatase activities (Fig. 7). A significant increase in the total enzyme activities of all these enzymes were found in the larvae fed on CanDef-20. Amylase, protease, GST and aminopeptidase showed an increase of around 50–63% whereas lipases and alkaline phosphatases showed 35% and 24% increase respectively in CanDef-20 fed larvae. The RNA seq data had indicated a prominent downregulation of most of the isoforms of genes for these enzymes in response to Candef-20 feeding (Fig. 6). However, a few specific enzyme isoform coding genes were found to be upregulated for all the selected enzymes (except amylases). These selectively upregulated enzyme isoforms coding genes would have most prominently contributed to the increase in net in vitro activity of these enzymes.

### Validation of selected differentially regulated *H. armigera* genes upon CanDef-20 ingestion by real-time quantitative reverse transcription PCR (RT-qPCR)

**Figure 8:**
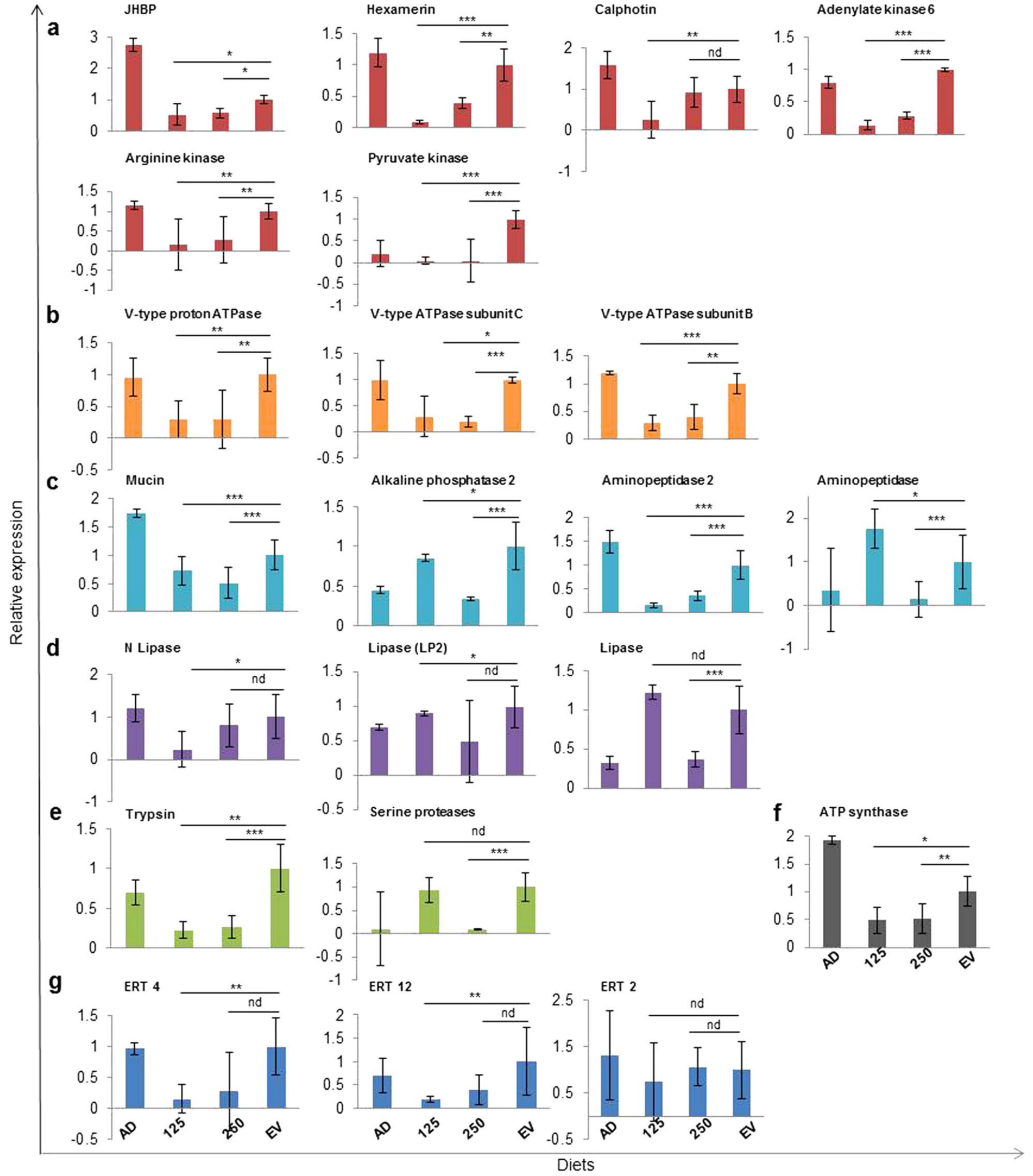
RNA-seq gene expression validation by qPCR analysis: qPCR analysis for selected genes is performed to validate the RNA-seq data. (**a**) Juvenile hormone binding protein (JHBP), hexamerin, calphotin, arginine kinase, pyruvate kinase, adenylate kinase 6, (**b**) V-type proton ATPase and its subunit C and B. (**c**) aminopeptidase 2, alkaline phosphatse 2 and aminopeptidase (**d**) N lipase, lipase (LP2), lipase. (**e**) trypsin, serine protease. (**f**) ATP synthase. (**g**) Endonuclease reverse transcriptase (ERT 4, ERT 12 and ERT 2). The relative gene expression values are significantly different from EV control at * for *P* < 0.05, ** for *P* < 0.01, *** for *P* < 0.001 and nd for no difference respectively.

Relative gene expression levels of JHBP, hexamerin, calphotin, kinases, ATPases, integral components of membrane, lipases, serine endopeptidases and transposons were studied from CanDef-20 fed *H. armigera* larvae (at 125 μg/ml and 250 μg/ml) and EV control by RT-qPCR (details of primer are given in Supplementary Table S3). The gene expression of JHBP, hexamerin and calphotin were found to be downregulated in *H. armigera* upon CanDef-20 ingestion and this is consistent with its RNA-seq data (Fig. 8a).

The isoforms of arginine kinase, pyruvate kinase, V-type ATPase and its subunit C, were down regulated and this is consistent with its RNA-seq data, but adenylate kinase and V-type ATPase subunit B showed contrasting expression levels in qPCR versus RNA-seq analysis (Fig. 8a,b).

Gene expression levels of isoforms of membrane proteins like, aminopeptidase, alkaline phosphatase 2 and mucin were also down regulated and it is consistent with RNA-seq data (Fig. 8c).

Different isoforms of lipases (lipase, N lipase and LP2), serine protease and trypsin were found to be up or down regulated and that is consistent with their respective RNA-Seq data (Fig. 8d and e).

Similarly, ATP synthase also showed down regulation of gene expression correlating with the RNA-seq data (Fig. 8f). qPCR analysis showed contrasting expression pattern of all the tested isoforms of endonuclease reverse transcriptase genes (ERT4, 12 and 2) with its RNA-seq data respectively (Fig. 8g).

## Discussion

Plants employ arsenal of biomolecules to pose a defense response against pests and pathogens, some of these molecules are target specific while some are broad acting. Plant defensins are best known for their antifungal properties^33^ though they also possess insect growth retardation potential. In the present study ingestion of a defensin from *C. annuum* (recombinant CanDef-20) led to a dose dependent negative effect on the growth and development of the insect pest *H. armigera*, similar to lepidopteran and coleopteran insect antibiosis reported earlier^34,35^. *H. armigera* fed very high concentrations of CanDef-20 (250 µg/ml) deviated from the trend of dose dependent larval mass reduction, most likely due to the defensin induced adaptive molecular responses (discussed further).

**Figure 9:**
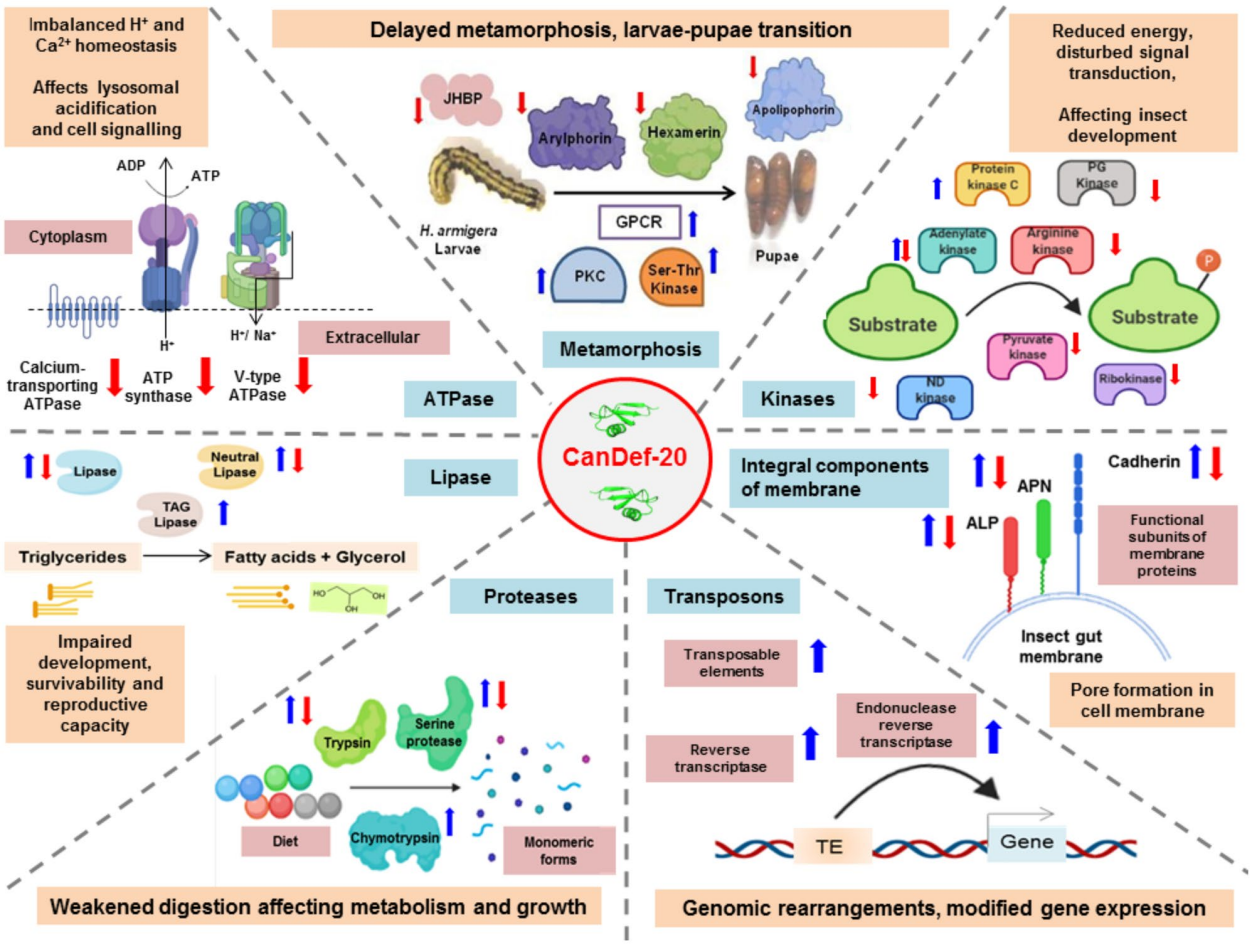
Model diagram representing metabolic reprogramming/pathways affected by CanDef-20 ingestion in *H. armigera*: Selected gene categories like transposons, kinase, integral components of membrane, serine endopeptidase, lipase, ATPase are shown and the role of key proteins involved in the process are highlighted. The down and upregulation of specific proteins and enzymes are shown by red and blue arrows respectively.

Though insect growth retardation is a prominent effect due to feeding on defensin containing diet, the molecular mechanisms involved in plant defensin-mediated metabolic changes in insects like delayed life stages, growth retardation, mass reduction and loss of fecundity remain largely unknown. The comparative transcriptomics revealed changes in pathways and processes induced by CanDef-20 ingestion in *H. armigera* larvae. AMPs like defensins are known to interact with different cellular components like cell membrane, cytoplasmic proteins and they can also enter the nucleus and interact with DNA^36–39^. Ingestion of CanDef-20 peptide mediates multiple effects on *H. armigera* metabolism through defensin–secreted protein/enzyme interaction(s), defensin-membrane interactions and defensin-induced regulation of target gene expression (Fig. 9).

One of the known modes for plant defensin activity on insects is in the form of inhibition of their digestive enzymes like amylases and proteinases^17,40–42^. In the present study, CanDef-20 fed larvae showed higher gut amylase and proteinase activities. Though, the transcriptomics data revealed a prominent downregulation of alpha-amylase and serine endopeptidase (serine proteases and trypsin) transcripts and notable upregulation of a few isoforms of chymotrypsin and serine protease. This indicated compensatory metabolic adjustments made by *H. armigera* for balancing digestion in presence of CanDef-20. Adjustments in the expression of proteinase isoforms at gene and transcript levels in insects are common upon exposure to proteinase inhibitors^43,44^. Similarly, gut amylase enzymes modulate their expression in response to ingestion of plant amylase inhibitor^45^.

The insect lipase inhibitory potential of plant defensins has not been reported earlier, though we noted a downregulation of most *H. armigera* lipase transcripts (neutral lipase, lipases and triacylglycerol lipases) upon CanDef-20 ingestion. Also, a simultaneous net increase in lipase activity in CanDef-20 fed larvae was observed, which can be attributed to the compensatory upregulated lipase transcript isoforms LP1, LP2 and TLP (classified under carboxylic ester hydrolase activity [GO:0052689]). Carboxylic ester hydrolases are also involved in the detoxification of several insecticides imparting resistance development in insects^46^. It is also known that different isoforms of lipase enzymes are needed for lipid mobilization in insect under starvation stress^47^. TAG lipases and a phospholipase gets upregulated during starvation in *D. melanogaster* and it may be involved in lipid mobilization^48,49^. Thus CanDef-20 ingestion seems to induce starvation-like stress and toxin stress in *H. armigera,* which further manifests into differential lipase expression and carboxylic ester hydrolase mediated lipid breakdown.

The available genomic and transcriptomic resources for *H. armigera* are not sufficient to map the range of transcripts expressed upon CanDef-20 ingestion as 64% of the differentially expressed transcripts identified in the present study are uncharacterized genes. The recently published *H. armigera* genome^50^ resource may be useful to annotate some of the uncharacterized genes from the present study.

Recent studies have shown that defensins interact with microbial pathogens and/or tumour cell membranes by binding to specific phospholipids to cause membrane permeabilization leading to cell death^38,51,52^. The mechanism of membrane disruption varies as per the membrane composition and interacting defensin molecule(s). The fungal membrane interaction kinetics has been known for two plant defensins, (i) pea defensin PsD1 interacting with membranes containing the sphingolipid glucosylceramide (GluCer)^53^ and (ii) MtDef4 from *Medicago truncatula* interacting with membrane phosphatidic acid (PA)^15^. We noted that integral components of the membrane (GO:0,016,021) is a highly downregulated GO in CanDef-20 fed *H. armigera* larvae, and it represents membrane proteins like V-type proton ATPases (V-ATPases), Cadherin, ALP and APN proteins.

V-ATPases transmembrane proton pumps which play important roles in numerous biological processes, such as protein degradation and synthesis, shifts in organelle pH, cell growth and cell autophagy^54^. CanDef-20 ingestion would lead to disturbance in cellular energy homeostasis in *H. armigera*, which can be correlated with the downregulation of V-ATPases. V-ATPases and its subunit were downregulated during starvation in tobacco hornworm *Manduca sexta*^55^. Interestingly, the RNAi targeting V-ATPase in *H. armigera* reduced larval mass and survival ability of the larvae^56^, correlating with the findings from the present work.

Cadherin, ALP and APN proteins are functional receptors for the Cry1A toxin in *Helicoverpa armigera* and their interaction with cry toxin mediates pore formation in the gut membrane leading to insect death^5^. Downregulation of these receptors and expression of their mutated isoforms leads to the development of resistance against Cry1A toxin in lepidopteran insects^4,57,58^. CanDef-20 ingestion-induced downregulation of Cadherin, ALP and APN receptors in *H. armigera* is indicative of the toxic effect of CanDef-20. Additionally, transcripts of mutant cadherin and some isoforms of ALP and APN are seen to be upregulated in *H. armigera* upon CanDef-20 ingestion, revealing similarities in processes triggered by Cry1A toxin and CanDef-20. Similar simultaneous downregulation of APN1 and compensatory upregulation of APN6 is reported in the Cry1A resistant cabbage looper, *Trichoplusia ni*^6^.

GSTs are the other enzymes with xenobiotic detoxification potential^59^. The downregulation of several isoforms of GSTs and selective upregulation of two GST isoforms upon CanDef-20 ingestion in *H. armigera* is also indicating the toxic stress of CanDef-20 and corresponding adaptive response of the insect, as also represented by high GST protein activity (Fig. 7).

CanDef-20 ingestion leads to a change in the expression of some of the cytoplasmic enzymes like Arginine kinase (AK), Pyruvate kinase (PK), Superoxide demutase (SOD), Spermine oxidase and Chitin synthase (CHS).

AK is an invertebrate phosphagen kinase involved in the transport and buffering of energy by stabilizing the cellular ATP/ADP ratio^60^. RNA interference (RNAi) study targeting AK in coleopteran beetle *Phyllotreta striolata* resulted in mortality and loss in fecundity^61^. PK catalyzes phosphoenol pyruvate to pyruvate reaction of glycolysis, leading to the TCA cycle^62^. The inhibition of PK leads to decreased ATP levels and an imbalance in the energy metabolism in *Tribolium castaneum*^63^. Low levels of pyruvate in the pupal brains of *H. armigera* resulted in an extension of lifespan by initiating diapause^64^. The reduced larval and pupal mass along with the delayed pupation in CanDef-20 fed *H. armigera* may be correlated with the downregulation of AK and PK.

Plant defensins can cause cellular effects including generation of reactive oxygen species (ROS), which triggers secondary signaling and imbalance of ionic homeostasis, ultimately contributing to cell death^65^. SOD enzyme helps in combating ROS generation^66^. Plant defensins like RsAFP2 and Vu-Defr are also capable to induce ROS in *Candida albicans*^67^ and *Leishmania amazonensis*^68^ respectively. Upregulation of SOD upon CanDef-20 ingestion in *H. armigera* implies that defensin is generating oxidative stress in the larvae and the elevated SOD expression relieves ROS stress.

Interference in the activity of ribosomal machinery may impact the growth and development of insects^69^. In *Drosophila*, the transcript level of ribosomes and protein synthesis is downregulated under starvation of dietary amino acids^70^. The decrease in the transcript level of ribosomes is the result of nutrient stress posed by CanDef-20 feeding by *H. armigera*.

CHS maintains the integrity of the chitin matrix and regulates larval to pupal metamorphosis, egg hatching and fecundity^71^. The silencing of CHS genes resulted in malformed phenotypes and increased mortality with decreased molting rate in the nymph of *Locusta migratoria manilensis*^72^ and egg laying in red mite *Panonychus citri*^73^. The extension of the larval period and decrease in egg laying may be a consequence of the downregulation of the CHS gene in CanDef-20 fed *H. armigera* larvae (Fig. 2).

Juvenile hormone binding protein (JHBP) in association with juvenile hormone (JH) and storage proteins like hexamerin, arylphorin, apolipophorin are essential for multiple physiological processes in insects including larval development, metamorphosis, and adult reproduction^74^. It is reported that down regulation of JHBP leads to developmental delays in *H. armigera* larvae^74^. Also, hexamerin was found to be significantly down-regulated in pine sawyer beetle (*Monochamus alternatus*) when fed with insecticides (chloramine phosphorus)^75^, and arylphorin was down-regulated in Vip3Aa toxin-treated larvae of *Spodoptera exigua*^76^. Thus, down regulation of JHBP, hexamerin and arylphorin might have impact on metamorphosis of *H. armigera* larvae leading to a prolonged larval stage. However, counter upregulated transcripts of protein kinase, serine-threonine kinase and G-protein receptors helps in proceeding for pupal stage.

Endonuclease reverse transcriptase (retrotransposons) and transposable element (TEs) were the most represented upregulated transcripts in CanDef-20 fed *H. armigera* larvae. TEs by way of insertion mutagenesis and recombination near the functional genes contribute to the generation of novel mutations and are responsible to generate much of the genetic diversity that contributes to rapid evolution^77^. Insertion of TEs near detoxification genes like P450 conferred the adaptation and resistance to insecticides in *H. zea* moths and *D. melanogaster* flies^78^. The transposon insertion in gut membrane receptor cadherin genes leads to Bt resistance in pink bollworm^79^. We observed that feeding with CanDef-20 triggered the mobilization of RNA-type TEs like LINE jockey, LTR gypsy and LINE RTE in *H. armigera*. *H. virescens* showed upregulation of retrotransposons and DNA transposons in response to ingestion of glucosinolates from *Arabidopsis thaliana*^80^. Increased expression and transposition of TEs in response to CanDef-20 may generate heritable genetic diversity in *H. armigera* larvae and confer further selective advantages to the insects, which needs further investigations.

Disturbance in metabolism of larval stage due to the presence of inhibitors or toxins in the diet often offsets its life cycle. Our findings demonstrate that CanDef-20 ingestion exhibited larval and pupal growth inhibition in *H. armigera* larvae. Comparative transcriptomics analysis indicated a downregulation and differential upregulation of genes involved in various metabolic pathways including energy homeostasis and enzymes related to food digestion, xenobiotic detoxification upon exposure to CanDef-20. We conclude that the retardation in the growth and development of *H. armigera* larvae ingesting CanDef-20 is mediated through its interactions with secreted proteins/enzymes, membrane components and regulation of target gene expression mediated via transcription factors and transposons. Our study provides a basis for future research on the potential practical applications of defensin peptide mediated control of the damaging pests like *H. armigera*.

## Supplementary Information

Supplementary Information 1.

Supplementary Information 2.

Supplementary Information 3.

Supplementary Information 4.

## Data Availability

The transcriptome assembly data generated during the current study is available at SRA (NCBI database) under the bioproject PRJNA869208. Additional data files on the transcriptome assembly are available at Mendeley data https://doi.org/10.17632/gwjdhw6rw3.1.
