# Supplementary material for: Novel insights into plant defensin ingestion induced metabolic responses in the polyphagous insect pest Helicoverpa armigera
Source: Sci Rep. 2023 Feb 23;13:3151. doi: 10.1038/s41598-023-29250-3 (PMC9950371; doi:10.1038/s41598-023-29250-3)
Supplement: Supplementary file 1 — Supplementary Information 1. [file 41598_2023_29250_MOESM1_ESM.docx]

**Novel insights into plant defensin ingestion induced metabolic responses in the polyphagous insect pest *Helicoverpa armigera***

Javed A. Mulla, Vaijayanti A. Tamhane*

Department of Biotechnology (jointly merged with Institute of Bioinformatics and Biotechnology (IBB)), Savitribai Phule Pune University, Pune, Maharashtra 411007, India.

**Corresponding Author**

Dr. Vaijayanti Tamhane

Assistant Professor

Department of Biotechnology

(Jointly merged with Institute of Bioinformatics and Biotechnology (IBB)),

Savitribai Phule Pune University,

Pune, Maharashtra 411007,

India

[vatamhane@unipune.ac.in](mailto:vatamhane@unipune.ac.in), [vatamhane@gmail.com](mailto:vatamhane@gmail.com)

Orcid: 0000-0001-5218-5359

**Supplementary Figure S1: The SDS-PAGE and dot blot analysis of recombinant CanDef-20 and EV proteins** a. CanDef-20 and other defensin protein were resolved on 15% SDS-PAGE and visualized by Coomassie blue staining. The number 1 and 2 denoted the recombinant proteins isolated after dialysis and total *Pichia pastoris* cellular proteins isolated by sonication in presence of lysis buffer respectively. b. The EV control protein with some other proteins were resolved on 15% SDS-PAGE and visualized by Coomassie blue staining. The protein molecular weight ladder (Genei Laboratories Private Limited) is shown in lane M. c. Dot blot analysis was performed for CanDef-20 and EV control proteins by using rabbit anti-myc primary antibody against myc epitope tag of pPICZ-alpha A vector. Secondary antibody provided with Western Blot Development kit (Bangalore genie, India) was used to visualize myc-tagged primary antibody complex.

**Supplementary Figure S2. Effect on growth and development of *H. armigera* larvae fed with CanDef-20, control (AD) and EV control diet** a and b. The larval and pupal mass of *H. armigera* larvae fed with CanDef-20, control (AD) and EV control diet are shown by median plot. The values are significantly different from EV control at * for p < 0.05, **for p < 0.01 and *** for p < 0.001, respectively. c. An independent *H. armigera* bioassay. *H. armigera* larvae fed with two different concentrations of CanDef-20 (150 and 300 µg/ml). The delayed pupation was more pronounced in the larvae fed with CanDef-20 (300 µg/ml).


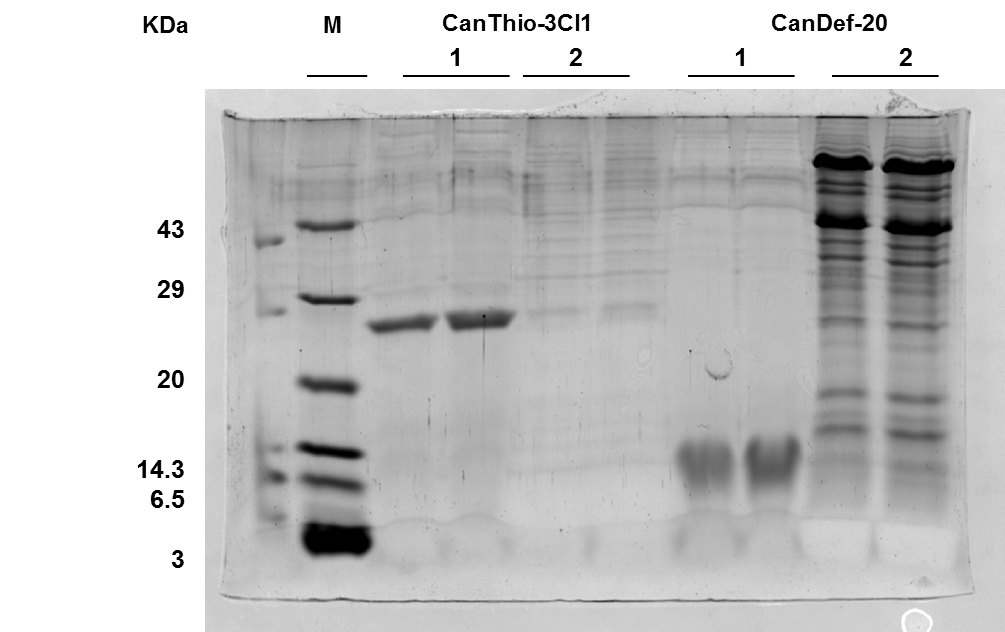


a

b


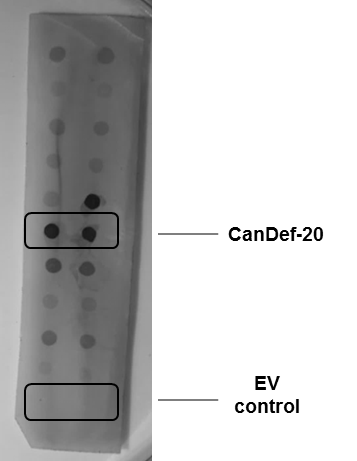


c


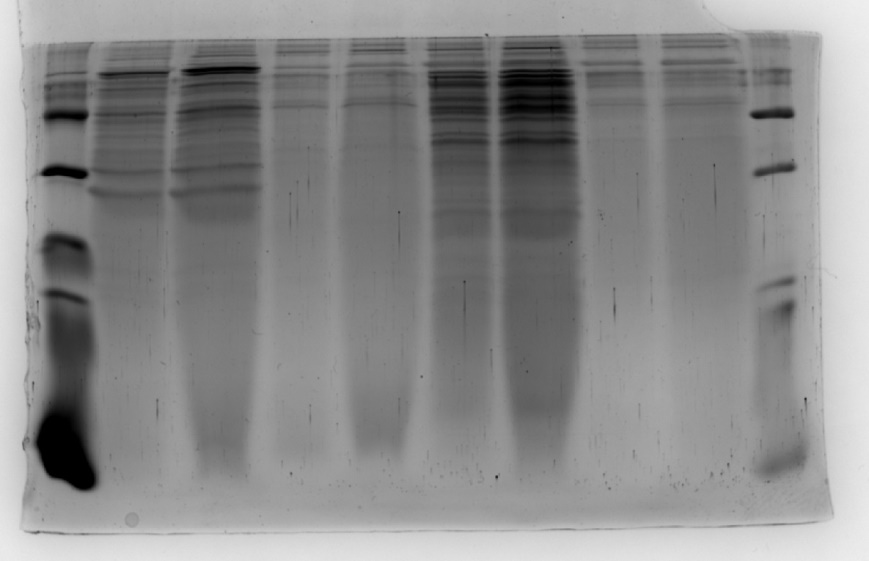


**KDa**

**EV**

**control**

**Other proteins**

**M**

**M**

**43**

**29**

**20**

**14.3**

**6.5**

**3**


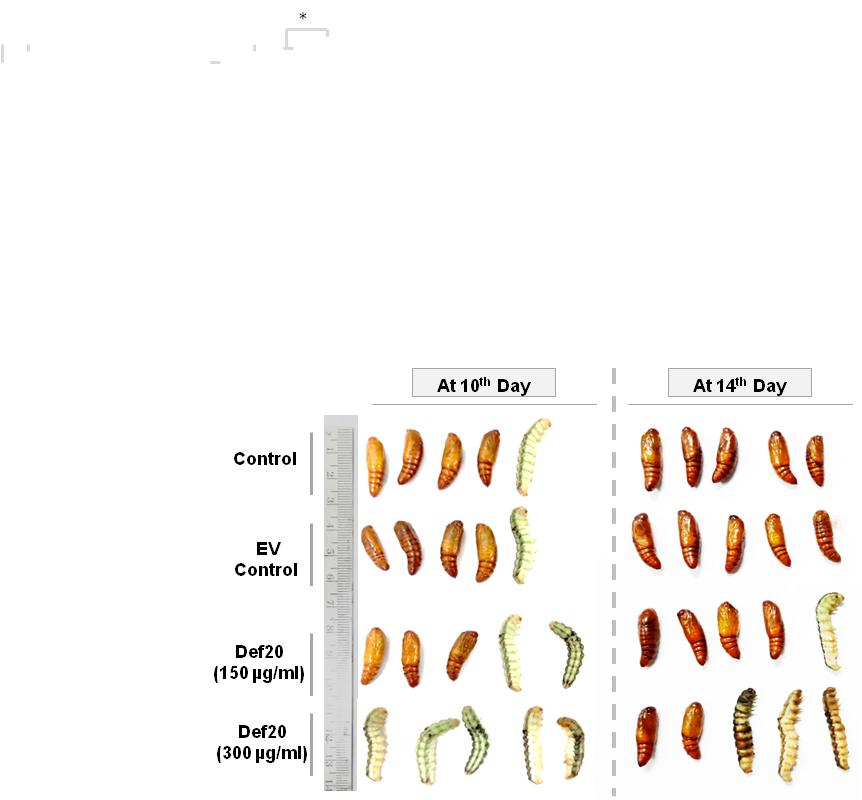

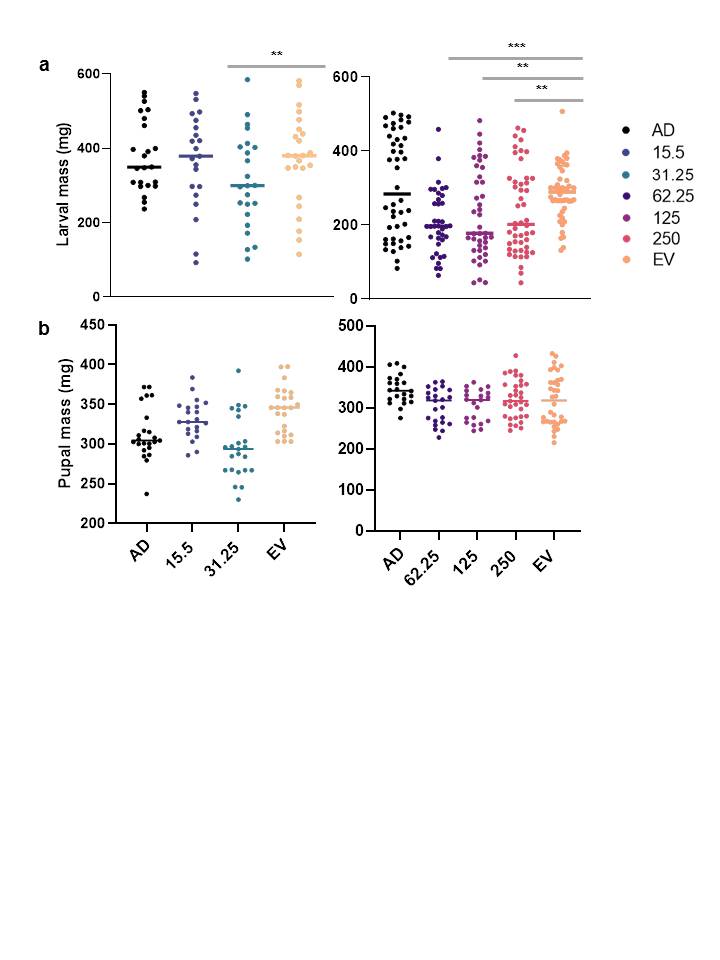


**c**

**Supplementary Figure S3: Gene Ontology enrichment of DEGs of CanDef-20 fed insects compared to EV control as per ≥2-fold criteria with P-value ≤0.05 and FDR value ≤0.05:** a. Gene Ontology (GO) classifications of transcripts into three categories like molecular function, cellular component and biological process. The X-axis corresponds to the number of DEGs appeared in analysis while the Y-axis represents different GOs. Red and blue bars are showing down and up regulation respectively.

**
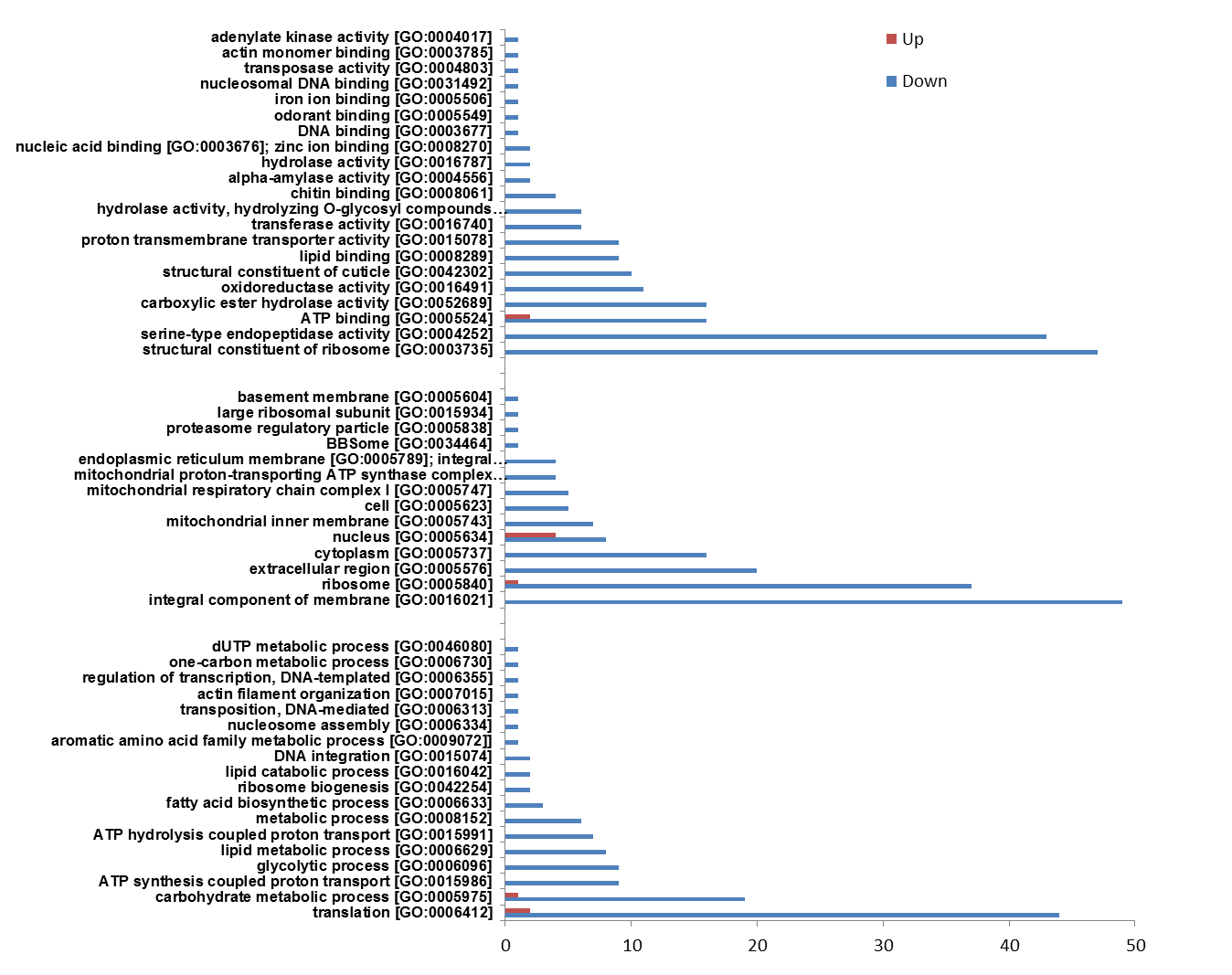
**

**
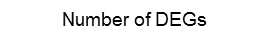
**

**Supplementary Table S1: Top 25 differentially expressed genes found in *H. armigera* larvae fed with CanDef-20 and EV control**

The top 25 genes fall into the categories into upregulated and unique detected in CanDef-20 fed larvae (treatment), downregulated and uniquely absent in CanDef-20 fed larvae (detected only in control) are shown (Based on log_2_FC ≥ +0.8 and **≤** -0.8 ratio).

| **Top 25 DEGs upregulated in CanDef-20 fed larvae** | | | | | | | | | | |
| --- | --- | --- | --- | --- | --- | --- | --- | --- | --- | --- |
| **Sr. No.** | **Transcript id** | | **FPKM Control** | | **FPKM Treated** | **Fold change** | **Matched Uniprot id** | **Protein name** | | **Organism name** |
| 1 | TRINITY_DN82260_c0_g1_i3 | | 0.02 | | 2.09 | 6.71 | A0A2A4IUH0_HELVI | Uncharacterized protein-  ([hypothetical protein B5X24_HaOG215920 [*H.armigera*]](https://blast.ncbi.nlm.nih.gov/Blast.cgi#alnHdr_PZC85650)) | | *Heliothis virescens* |
| 2 | TRINITY_DN142822_c0_g1_i1 | | 2 | | 124 | 5.95 | A0A2A4K8T2_HELVI | Uncharacterized protein  ([polyhomeotic-like protein 2 [*H. armigera*]](https://blast.ncbi.nlm.nih.gov/Blast.cgi#alnHdr_XP_021181147) 96% 84.4%) | | *Heliothis virescens* |
| 3 | TRINITY_DN82468_c1_g1_i2 | | 0.11 | | 6.43 | 5.87 | B4Z1D7_HELAM | Alkaline phosphatase (EC 3.1.3.1) | | *Helicoverpa armigera* |
| 4 | TRINITY_DN82736_c2_g5_i1 | | 3 | | 158 | 5.72 | A0A2A4JEL6_HELVI | Uncharacterized protein  ([fibroin heavy chain-like [*H. armigera*]](https://blast.ncbi.nlm.nih.gov/Blast.cgi#alnHdr_XP_021192154) 100% 93.10%) | | *Heliothis virescens* |
| 5 | TRINITY_DN82035_c0_g1_i2 | | 1 | | 35.91 | 5.17 | A0A2A4IY53_HELVI | Uncharacterized protein  ([fibroin heavy chain-like isoform X1 [*H. armigera*]](https://blast.ncbi.nlm.nih.gov/Blast.cgi#alnHdr_XP_021195533) 100% 91.78%) | | *Heliothis virescens* |
| 6 | TRINITY_DN75316_c0_g1_i1 | | 1 | | 27 | 4.75 | A0A0L7KW86_9NEOP | Putative pol polyprotein | | *Operophtera brumata* |
| 7 | TRINITY_DN83412_c6_g2_i1 | | 5.57 | | 132.62 | 4.57 | A0A2H1WG43_SPOFR | SFRICE_037529 (Fragment)-  (endonuclease-reverse transcriptase [*Bombyx mori*] 100% 79.29%) | | *Spodoptera frugiperda* |
| 8 | TRINITY_DN93531_c0_g1_i1 | | 1 | | 20 | 4.32 | A0A2A4J358_HELVI | Uncharacterized protein  ([mitochondrial potassium channel-like [*S. frugiperda*]](https://blast.ncbi.nlm.nih.gov/Blast.cgi#alnHdr_XP_035456344)) 100% 82.87% | | *Heliothis virescens* |
| 9 | TRINITY_DN82493_c0_g1_i1 | | 1 | | 18 | 4.17 | A0A2A4JAI1_HELVI | Uncharacterized protein  ([uncharacterized protein LOC110378134 [*H. armigera*]](https://blast.ncbi.nlm.nih.gov/Blast.cgi#alnHdr_XP_021192938) 100% 77.71% | | *Heliothis virescens* |
| 10 | TRINITY_DN82325_c0_g1_i2 | | 1 | | 16.5 | 4.04 | A0A2A4K9W7_HELVI | Uncharacterized protein  ([neurofilament heavy polypeptide-like isoform X1 [*H.armigera*]](https://blast.ncbi.nlm.nih.gov/Blast.cgi#alnHdr_XP_021185043))100% 90.61% | | *Heliothis virescens* |
| 11 | TRINITY_DN81966_c0_g1_i1 | | 1.05 | | 15.85 | 3.92 | A0A2A4JT65_HELVI | Uncharacterized protein  ([troponin T, skeletal muscle isoform X1 [*H. armigera*]](https://blast.ncbi.nlm.nih.gov/Blast.cgi#alnHdr_XP_021187446)) 99% 99.23% | | *Heliothis virescens* |
| 12 | TRINITY_DN83041_c4_g5_i4 | | 2 | | 29 | 3.86 | A0A2H1W1U7_SPOFR | SFRICE_026509 (Fragment)  reverse transcriptase [*O. brumata*] 58% 36.31% | | *Spodoptera frugiperda* |
| 13 | TRINITY_DN83038_c7_g1_i11 | | 1 | | 13.86 | 3.79 | D1LU95_HELAM | Mutant cadherin | | *Helicoverpa armigera* |
| 14 | TRINITY_DN22204_c0_g2_i1 | | 1 | | 13 | 3.70 | A0A0N1PFX5_PAPMA | Putative nuclease HARBI1 (Fragment) | | *Papilio machaon* |
| 15 | TRINITY_DN86374_c0_g1_i1 | | 1 | | 13 | 3.70 | A0A2H1WPU0_SPOFR | SFRICE_025439  ATP-binding cassette sub-family G member 4-like isoform X1 [*H. armigera*] 100% 98.79% | | *Spodoptera frugiperda* |
| 16 | TRINITY_DN83412_c6_g5_i1 | | 3 | | 35 | 3.54 | D7F157_BOMMO | Endonuclease-reverse transcriptase | | *Bombyx mori* |
| 17 | TRINITY_DN44683_c0_g1_i1 | | 1 | | 11 | 3.46 | H9JN76_BOMMO | Uncharacterized protein | | *Bombyx mori* |
| 18 | TRINITY_DN81365_c0_g1_i2 | | 1 | | 11 | 3.46 | A0A2A4J4L3_HELVI | Peptidyl-prolyl cis-trans isomerase E (PPIase E) (EC 5.2.1.8) | | *Heliothis virescens* |
| 19 | TRINITY_DN82601_c0_g2_i1 | | 2.38 | | 24 | 3.33 | A0A2H1VMJ9_SPOFR | SFRICE_000949  Transposable element Tc3 transposase-like Protein [*T. castaneum*] 97% 48.15% | | *Spodoptera frugiperda* |
| 20 | TRINITY_DN152499_c0_g1_i1 | | 1 | | 10 | 3.32 | A0A2A4J222_HELVI | Uncharacterized protein  hypoxia up-regulated protein 1 [*H. armigera*] 100% 98.37% | | *Heliothis virescens* |
| 21 | TRINITY_DN36008_c0_g1_i1 | | 1 | | 9 | 3.17 | A0A2A4J6C6_HELVI | Uncharacterized protein  formin-like protein [*S. frugiperda*] 70% 52.96% | | *Heliothis virescens* |
| 22 | TRINITY_DN82738_c4_g6_i1 | | 1 | | 9 | 3.17 | A0A2A4IYL5_HELVI | Uncharacterized protein (Fragment)  Zinc finger DNA binding protein [*O. brumata*] 98% 58.27% | | *Heliothis virescens* |
| 23 | TRINITY_DN113323_c0_g1_i1 | | 1 | | 9 | 3.17 | A0A2A4K8T2_HELVI | Un.characterized protein  polyhomeotic-like protein 2 [*H. armigera*] 96% 84.41% | | *Heliothis virescens* |
| 24 | TRINITY_DN12026_c0_g1_i1 | | 1 | | 9 | 3.17 | G9LPP2_HELAM | UDP-glucuronosyltransferase (EC 2.4.1.17) | | *Helicoverpa armigera* |
| 25 | TRINITY_DN82714_c3_g4_i3 | | 1.72 | | 15.25 | 3.15 | A0A2H1VRU3_SPOFR | SFRICE_019335  Retrovirus-related Pol polyprotein from type-2 retrotransposable element R2DM [*F. candida*] 95% 49.04% | | *Spodoptera frugiperda* |
| **Unique detected in CanDef-20 fed larvae (treatment)** | | | | | | | | | | |
| **Sr. No.** | **Transcript id** | | **FPKM Control** | | **FPKM Treated** | **Fold change** | **Matched Uniprot id** | **Protein name** | **Organism name** | |
| 1 | TRINITY_DN82097_c0_g1_i2 | | 0 | | 211.99 | - | A0A2A4JHS8_HELVI | Uncharacterized protein  Arginine kinase isoform X2 [*H. armigera*] 100% 99.21% | *Heliothis virescens* | |
| 2 | TRINITY_DN83424_c7_g1_i5 | | 0 | | 123.76 | - | D7F159_BOMMO | Endonuclease-reverse transcriptase | *Bombyx mori* | |
| 3 | TRINITY_DN81719_c0_g1_i1 | | 0 | | 114.71 | - | A0A0K8TIS0_LYGHE | Uncharacterized protein (Fragment)  putative G-protein coupled receptor Mth-like 2 [*P. xuthus*] 51% 49.15% | *Lygus hesperus* | |
| 4 | TRINITY_DN83424_c7_g1_i2 | | 0 | | 110.41 | - | D7F159_BOMMO | Endonuclease-reverse transcriptase | *Bombyx mori* | |
| 5 | TRINITY_DN83386_c1_g3_i10 | | 0 | | 100.83 | - | O18447_HELAM | Serine protease (Trypsin-like protease) | *Helicoverpa armigera* | |
| 6 | TRINITY_DN82869_c6_g1_i7 | | 0 | | 83.12 | - | F8SL48_HELAM | Parathyroid hormone-responsive B1 | *Helicoverpa armigera* | |
| 7 | TRINITY_DN83028_c3_g1_i5 | | 0 | | 75 | - | A0A2A4JBB2_HELVI | ATP synthase subunit gamma | *Heliothis virescens* | |
| 8 | TRINITY_DN82567_c9_g1_i1 | | 0 | | 49 | - | A0A2A4J5T2_HELVI | Uncharacterized protein (Fragment)  eukaryotic translation initiation factor 5B [*H.armigera*] 100% 97.65% | *Heliothis virescens* | |
| 9 | TRINITY_DN60361_c0_g1_i1 | | 0 | | 33 | - | A0A2H1W6I0_SPOFR | SFRICE_007352  GPI ethanolamine phosphate transferase 3-like isoform X2 [*S. frugiperda*] 100% 99.7% | *Spodoptera frugiperda* | |
| 10 | TRINITY_DN82325_c0_g1_i1 | | 0 | | 32.07 | - | A0A2A4K9W7_HELVI | Uncharacterized protein  Replicase large subunit [*O. brumata*] 97% 64.4% | *Heliothis virescens* | |
| 11 | TRINITY_DN83167_c2_g2_i7 | | 0 | | 28.63 | - | A0A2A4JI81_HELVI | Uncharacterized protein  regucalcin-like isoform X1 [*H. armigera*] 100% 94.74% | *Heliothis virescens* | |
| 12 | TRINITY_DN83395_c5_g1_i3 | | 0 | | 25 | - | A0A2A4JSE8_HELVI | Uncharacterized protein  Replicase large subunit [*O. brumata*]97% 64.4% | *Heliothis virescens* | |
| 13 | TRINITY_DN82343_c0_g1_i6 | | 0 | | 24.58 | - | A0A2H1VN30_SPOFR | SFRICE_000249  Paramyosin, short form [*O. brumata*] 99% 80.41% | *Spodoptera frugiperda* | |
| 14 | TRINITY_DN82713_c0_g1_i11 | | 0 | | 22.99 | - | A0A2A4JBU6_HELVI | Uncharacterized protein  activity-regulated cytoskeleton associated protein 1-like [*M. sexta*] 84% 43.92% | *Heliothis virescens* | |
| 15 | TRINITY_DN82410_c0_g1_i1 | | 0 | | 22.47 | - | A0A2A4K5I8_HELVI | Uncharacterized protein  translation elongation factor 2 [*H. armigera*] 100% 99.64% | *Heliothis virescens* | |
| 16 | TRINITY_DN82716_c2_g1_i2 | | 0 | | 22.22 | - | A0A168T0D5_HELAM | Juvenile hormone binding protein 1 | *Helicoverpa armigera* | |
| 17 | TRINITY_DN82035_c0_g1_i1 | | 0 | | 21.52 | - | A0A2A4IY53_HELVI | Uncharacterized protein  fibroin heavy chain-like isoform X1 [*H. armigera*] 100% 91.78% | *Heliothis virescens* | |
| 18 | TRINITY_DN82818_c1_g2_i1 | | 0 | | 20.47 | - | A0A2H1VMX5_SPOFR | SFRICE_016013  PREDICTED: RNA-directed DNA polymerase from mobile element jockey-like [*A. transitella*] 46% 42% | *Spodoptera frugiperda* | |
| 19 | TRINITY_DN82776_c10_g4_i9 | | 0 | | 20.12 | - | A0A2A4K3P0_HELVI | Uncharacterized protein  chitin deacetylase 1-like isoform X1 [*S. frugiperda*] 100% 99.26% | *Heliothis virescens* | |
| 20 | TRINITY_DN82478_c0_g1_i3 | | 0 | | 20 | - | A0A2A4J065_HELVI | Uncharacterized protein  glutenin, high molecular weight subunit PW212-like [*S frugiperda*] 99% 63.39% | *Heliothis virescens* | |
| 21 | TRINITY_DN82343_c0_g1_i5 | | 0 | | 19.95 | - | I4DIM8_PAPXU | Paramyosin | *Papilio xuthus* | |
| 22 | TRINITY_DN80813_c0_g1_i1 | | 0 | | 19.43 | - | A0A2A4K6D7_HELVI | Adenosyl homocysteinase (EC 3.3.1.1) | *Heliothis virescens* | |
| 23 | TRINITY_DN83179_c0_g1_i1 | | 0 | | 18.37 | - | A0A2A4K0J9_HELVI | Uncharacterized protein (Fragment)  Retrovirus-related Pol polyprotein from transposon 17.6 [*A. ventricosus*] 96% 33.56% | *Heliothis virescens* | |
| 24 | TRINITY_DN80989_c0_g2_i1 | | 0 | | 18 | - | A0A2A4J9P8_HELVI | Proteasome subunit alpha type (EC 3.4.25.1) | *Heliothis virescens* | |
| 25 | TRINITY_DN82367_c0_g1_i4 | | 0 | | 18 | - | B6CME5_HELAM | Chitin deacetylase (Fragment) | *Helicoverpa armigera* | |
| **Top 25 DEGs downregulated in CanDef-20 fed larvae** | | | | | | | | | | |
| **Sr. No.** | **Transcript id** | | **FPKM Control** | | **FPKM Treated** | **Fold change** | **Matched Uniprot id** | **Protein name** | **Organism name** | |
| 1 | TRINITY_DN82097_c0_g1_i1 | | 5524 | | 0.01 | -19.08 | A0A2A4JHS8_HELVI | Uncharacterized protein  Arginine kinase isoform X2 [*H. armigera*] 100% 99.21% | *Heliothis virescens* | |
| 2 | TRINITY_DN83296_c10_g1_i8 | | 1417.31 | | 0.01 | -17.11 | I4DLU5_PAPPL | Troponin I | *Papilio polytes* | |
| 3 | TRINITY_DN82109_c0_g1_i4 | | 90.7 | | 0.01 | -13.15 | I3QC09_HELAM | Lipase | *Helicoverpa armigera* | |
| 4 | TRINITY_DN82625_c1_g2_i2 | | 4318.53 | | 0.78 | -12.43 | A0A0K8TDX9_LYGHE | Uncharacterized protein (Fragment)  larval cuticle protein 1-like [*H. armigera*] 100% 98.37% | *Lygus hesperus* | |
| 5 | TRINITY_DN82136_c0_g2_i1 | | 1519.68 | | 0.78 | -10.93 | A0A2A4K1R5_HELVI | Uncharacterized protein  40S ribosomal protein S5 [*H. armigera*] 100% 99.54% | *Heliothis virescens* | |
| 6 | TRINITY_DN83198_c0_g1_i2 | | 11641.36 | | 6 | -10.92 | A0A2A4JMW0_HELVI | Uncharacterized protein  basic juvenile hormone-suppressible protein 1 [*H. armigera*] 97% 92.63% | *Heliothis virescens* | |
| 7 | TRINITY_DN83314_c0_g1_i12 | | 2526.5 | | 1.31 | -10.91 | A0A0K8SZQ3_LYGHE | Uncharacterized protein (Fragment)  pancreatic triacylglycerol lipase-like [*H. armigera*] 100% 100% | *Lygus hesperus* | |
| 8 | TRINITY_DN82610_c0_g1_i3 | | 1367.38 | | 0.87 | -10.62 | A0A2A4J2Y0_HELVI | Uncharacterized protein  calphotin-like [*H. armigera*] 98% 79.87% | *Heliothis virescens* | |
| 9 | TRINITY_DN82746_c0_g1_i1 | | 19580 | | 13 | -10.56 | Q68YP2_HELAM | Hexamerine | *Helicoverpa armigera* | |
| 10 | TRINITY_DN83202_c0_g3_i3 | | 4528.91 | | 3.58 | -10.30 | A0A0K8SDP7_LYGHE | Uncharacterized protein  calphotin-like [*H. armigera*] 100% 96.26% | *Lygus hesperus* | |
| 11 | TRINITY_DN82914_c0_g1_i6 | | 1853.85 | | 1.74 | -10.06 | B6CME3_HELAM | Insect intestinal mucin 2 (Fragment) | *Helicoverpa armigera* | |
| 12 | TRINITY_DN83234_c0_g1_i6 | | 45076.8 | | 57.52 | -9.61 | A0A2A4J4S9_HELVI | Uncharacterized protein  arylphorin subunit beta-like [*H. armigera*] 100% 96.60% | *Heliothis virescens* | |
| 13 | TRINITY_DN83314_c0_g3_i4 | | 760.6 | | 1 | -9.57 | I3QC11_HELAM | Neutral lipase | *Helicoverpa armigera* | |
| 14 | TRINITY_DN82574_c0_g1_i1 | | 461.92 | | 0.63 | -9.52 | A0A2A4JSL9_HELVI | Uncharacterized protein  gelsolin-like isoform X1 [*H. armigera*] 96% 91.73% | *Heliothis virescens* | |
| 15 | TRINITY_DN83234_c0_g1_i2 | | 2914.64 | | 4.06 | -9.49 | G3LF43_HELAM | Arylphorin | *Helicoverpa armigera* | |
| 16 | TRINITY_DN82063_c0_g1_i6 | | 6944.55 | | 11 | -9.30 | A0A0K8T7D1_LYGHE | Uncharacterized protein  actin cytoskeleton-regulatory complex protein PAN1-like [*H. armigera*] 100% 87.14% | *Lygus hesperus* | |
| 17 | TRINITY_DN82625_c1_g2_i1 | | 1585.35 | | 2.52 | -9.30 | A0A0K8TDX9_LYGHE | Uncharacterized protein (Fragment)  larval cuticle protein 1-like [*H. armigera*] 100% 98.37% | *Lygus hesperus* | |
| 18 | TRINITY_DN83154_c2_g1_i15 | | 897.3 | | 1.82 | -8.95 | A0A1B0RHP3_HELZE | Serine protease 3 (Fragment) | *Helicoverpa zea* | |
| 19 | TRINITY_DN82774_c9_g1_i12 | | 910.6 | | 1.85 | -8.94 | A0A0K8TJN2_LYGHE | Uncharacterized protein  Repat39-like protein [*H. virescens*] 95% 87.59% | *Lygus hesperus* | |
| 20 | TRINITY_DN83386_c1_g3_i11 | | 2548.21 | | 5.52 | -8.85 | O18447_HELAM | Serine protease  (Trypsin-like protease) | *Helicoverpa armigera* | |
| 21 | TRINITY_DN82805_c0_g1_i4 | | 9460.12 | | 24.71 | -8.58 | O18439_HELAM | Diverged serine protease | *Helicoverpa armigera* | |
| 22 | TRINITY_DN83191_c1_g1_i1 | | 356.11 | | 1 | -8.48 | I3QC07_HELAM | Neutral lipase | *Helicoverpa armigera* | |
| 23 | TRINITY_DN82625_c1_g1_i1 | | 9680 | | 28 | -8.43 | CULP1_HELAM | Larval cuticle protein 1 | *Helicoverpa armigera* | |
| 24 | TRINITY_DN83202_c0_g3_i2 | | 9989.16 | | 29.73 | -8.39 | A0A0K8SDP7_LYGHE | Uncharacterized protein  calphotin-like [*H. armigera*] 100% 96.26% | *Lygus hesperus* | |
| 25 | TRINITY_DN81221_c0_g1_i3 | | 773.93 | | 2.33 | -8.38 | A0A0F6Q0X5_HELAU | Alcohol dehydrogenase 6 (Fragment) | *Helicoverpa assulta* | |
| **Uniquely absent in CanDef-20 fed larvae (detected only in control)** | | | | | | | | | | |
| **Sr. No.** | | **Transcript id** | | **FPKM Control** | **FPKM Treated** | **Fold change** | **Matched Uniprot id** | **Protein name** | **Organism name** | |
| 1 | | TRINITY_DN83198_c0_g1_i1 | | 6054.64 | 0 | - | A0A2A4JMW0_HELVI | Uncharacterized protein  basic juvenile hormone-suppressible protein 1 [*H. armigera*] 97% 92.63% | *Heliothis virescens* | |
| 2 | | TRINITY_DN82657_c3_g1_i2 | | 1965.51 | 0 | - | A0A2A4K1V9_HELVI | Uncharacterized protein  serine protease snake-like [*H. armigera*] 100% 80.31% | *Heliothis virescens* | |
| 3 | | TRINITY_DN82538_c0_g1_i4 | | 1386.38 | 0 | - | A0A0D3M5W3_HELAM | Glutathione S-transferase GSTS2 | *Helicoverpa armigera* | |
| 4 | | TRINITY_DN83020_c0_g1_i2 | | 1346.71 | 0 | - | A0A2A4JJH0_HELVI | Uncharacterized protein  aldehyde dehydrogenase X, mitochondrial-like [*H. armigera*] 100% 94.88% | *Heliothis virescens* | |
| 5 | | TRINITY_DN81087_c0_g1_i5 | | 1221.25 | 0 | - | RS14_BOMMO | 40S ribosomal protein S14 | *Bombyx mori* | |
| 6 | | TRINITY_DN81966_c0_g1_i4 | | 1127.22 | 0 | - | A0A2A4JRD0_HELVI | Uncharacterized protein  troponin T, skeletal muscle isoform X3 [*H. armigera*] 99% 99.22% | *Heliothis virescens* | |
| 7 | | TRINITY_DN82748_c9_g1_i3 | | 880.8 | 0 | - | B1NLE0_HELAM | Uncharacterized protein  27 kDa hemolymph protein-like [*H. armigera*] 100% 100% | *Helicoverpa armigera* | |
| 8 | | TRINITY_DN83060_c6_g2_i2 | | 725.71 | 0 | - | B1NLD6_HELAM | Chitin binding PM protein | *Helicoverpa armigera* | |
| 9 | | TRINITY_DN81845_c0_g1_i1 | | 683.75 | 0 | - | A0A2A4JHC9_HELVI | Uncharacterized protein  ATP synthase subunit delta, mitochondrial [*H. armigera*] 100% 100% | *Heliothis virescens* | |
| 10 | | TRINITY_DN81966_c0_g1_i3 | | 677.52 | 0 | - | A0A2A4JT65_HELVI | Uncharacterized protein  troponin T, skeletal muscle isoform X1 [*H. armigera*]99% 99.23% | *Heliothis virescens* | |
| 11 | | TRINITY_DN83314_c0_g1_i9 | | 657.88 | 0 | - | A0A2A4K5B8_HELVI | Uncharacterized protein  pancreatic triacylglycerol lipase-like [*S. litura*] 100% 86.53% | *Heliothis virescens* | |
| 12 | | TRINITY_DN79583_c0_g2_i2 | | 605.87 | 0 | - | A0A2A4JKD0_HELVI | Uncharacterized protein  C-1-tetrahydrofolate synthase, cytoplasmic isoform X1 [*H. armigera*] | *Heliothis virescens* | |
| 13 | | TRINITY_DN83210_c1_g1_i6 | | 583.45 | 0 | - | I3QC06_HELAM | Neutral lipase | *Helicoverpa armigera* | |
| 14 | | TRINITY_DN81801_c0_g1_i1 | | 556.37 | 0 | - | A0A0K8S5N4_LYGHE | Uncharacterized protein (Fragment)  vegetative cell wall protein gp1-like [*H. armigera*] 93% 98.54% | *Lygus hesperus* | |
| 15 | | TRINITY_DN83112_c0_g2_i14 | | 542.09 | 0 | - | C7SB25_HELAM | Trypsin 2 | *Helicoverpa armigera* | |
| 16 | | TRINITY_DN83028_c3_g1_i1 | | 518.29 | 0 | - | A0A2A4JBB2_HELVI | ATP synthase subunit gamma | *Heliothis virescens* | |
| 17 | | TRINITY_DN82859_c0_g1_i2 | | 458.55 | 0 | - | B1NLE4_HELAM | Protease | *Helicoverpa armigera* | |
| 18 | | TRINITY_DN82384_c0_g1_i1 | | 452.65 | 0 | - | A0A2A4JFY8_HELVI | Uncharacterized protein  N-acetylneuraminate lyase-like [*H. armigera*] 100% 94.72% | *Heliothis virescens* | |
| 19 | | TRINITY_DN83172_c0_g1_i1 | | 445.65 | 0 | - | O96696_HELVI | Calcium-transporting ATPase (EC 3.6.3.8) | *Heliothis virescens* | |
| 20 | | TRINITY_DN82678_c0_g1_i1 | | 428.76 | 0 | - | A0A2A4K6Q0_HELVI | Uncharacterized protein | *Heliothis virescens* | |
| 21 | | TRINITY_DN82848_c11_g1_i2 | | 427.8 | 0 | - | A0A2A4JCC1_HELVI | Uncharacterized protein  40S ribosomal protein S16 isoform X1 [*H. armigera*] 100% 100% | *Heliothis virescens* | |
| 22 | | TRINITY_DN77494_c0_g1_i2 | | 410.61 | 0 | - | A0A2A4JIN5_HELVI | Uncharacterized protein | *Heliothis virescens* | |
| 23 | | TRINITY_DN82410_c0_g1_i2 | | 393.88 | 0 | - | A0A2A4K5I8_HELVI | Uncharacterized protein  translation elongation factor 2 [*H. armigera*]100% 99.64% | *Heliothis virescens* | |
| 24 | | TRINITY_DN82378_c0_g1_i3 | | 388.14 | 0 | - | A0A2A4JU62_HELVI | Uncharacterized protein  insecticyanin-A-like [*H. armigera*] 100% 95.35% | *Heliothis virescens* | |
| 25 | | TRINITY_DN82634_c12_g1_i2 | | 349.28 | 0 | - | A0A0K8T9K1_LYGHE | Uncharacterized protein (Fragment)  prion-like-(Q/N-rich) domain-bearing protein 25 [*G. mellonella*] 95% 44.57% | *Lygus hesperus* | |
