# Supplementary material for: Novel insights into plant defensin ingestion induced metabolic responses in the polyphagous insect pest Helicoverpa armigera
Source: Sci Rep. 2023 Feb 23;13:3151. doi: 10.1038/s41598-023-29250-3 (PMC9950371; doi:10.1038/s41598-023-29250-3)
Supplement: Supplementary file 3 — Supplementary Information 3. [file 41598_2023_29250_MOESM3_ESM.docx]

**Supplementary Table S3: List of primer used in RT-qPCR analysis**

| Sr. No. | Name of the gene | Transcript id | Forward primer | Reverse primer |
| --- | --- | --- | --- | --- |
| 1 | Juvenile hormone binding protein | TRINITY_DN83198_c0_g1_i2 | AGGACGACCTCACCTACCTC | CAGAGTGTTCACCGACGAGT |
| 2 | Hexamerin | TRINITY_DN82746_c0_g1_i1 | CGAAATCGAGGTTCCCCACA | AGTTCATCAGCCCTTGCTTCA |
| 3 | Calphotin | TRINITY_DN83202_c0_g3_i3 | AACGCTAACACCCCCGT | GTTCCTCAGGGAGGATGACG |
| 4 | Arginine kinase | TRINITY_DN82097_c0_g1_i1 | TTTGTGTCTTGCAGAAAAGCC | TAAGAGCGTCGAACACCTCC |
| 5 | Pyruvate kinase | TRINITY_DN81894_c0_g1_i1 | CGGACGGTATCATGGTCGCT | TCTGGGTGGCGCAGATTAC |
| 6 | Adenylate kinase | TRINITY_DN83366_c1_g1_i14 | ATCTAGCGCCAACTCCTGAAC | ACATCGTGGAAGGGAAGGTC |
| 7 | V-type proton ATPase | TRINITY_DN81493_c0_g1_i1 | GGTGTAGTTGTTGGAGGGGG | GTCCATCATTCCCGTCGTCA |
| 8 | V-type proton ATPase subunit C | TRINITY_DN82583_c10_g1_i2 | GCAACGCCTTGACGTGTATC | AGCTCGTCACCGACAAGAAG |
| 9 | V-type proton ATPase subunit B | TRINITY_DN82751_c6_g4_i1 | ACGCAAATACCCTAAGATCAGGAA | AAGTACCCATCCTAGCTTTGCC |
| 10 | Aminopeptidase 2 | TRINITY_DN83135_c1_g1_i1 | TGCGTCCATGGGTATATTGC | TCAAGCTGGCCTGGTTAAGAG |
| 11 | Alkaline phosphatase 2 | TRINITY_DN80034_c1_g2_i2 | GCGAGAAAAGACCCCAGTGA | AATGGCTACATCGCCACCTC |
| 12 | Aminopeptidase | TRINITY_DN83135_c1_g1_i5 | AACCGTTTCATTGTTTACAGGG | TCCAAACCACATGTGAGCGA |
| 13 | N lipase | TRINITY_DN83210_c1_g1_i6 | GAGTCGGTCACTGCTGAAGG | TGCAGGAGCGTCTTTTTCTCC |
| 14 | Lipase (LP2) | TRINITY_DN82609_c8_g2_i12 | AAACCCGCTGGCTCCATTC | CTGGGGCTAATGCCAACTTTC |
| 15 | Lipase | TRINITY_DN99909_c0_g1_i1 | TAGGCTCGACTGTGAGAGCA | ACGCAGGAGCTTATGTGGAG |
| 16 | Trypsin | TRINITY_DN83112_c0_g2_i14 | CTATCGGGTTGGGGAAGTCG | GTACGCAATCGCACAAGCAA |
| 17 | Serine proteases | TRINITY_DN83386_c1_g3_i10 | GTTACAAATACTTTTGCAGTGGCG | TGAGTTGTATGAGGGGTGGA |
| 18 | ATP synthase | TRINITY_DN81845_c0_g1_i1 | GTAGTCAAGGGGTGCCGAAA | GACTTGCAGCTTCCTAGCCA |
| 19 | Endonuclease reverse transcriptase (ERT4) | TRINITY_DN83232_c1_g1_i1 | GTCTCTGTTGGAAGCTCGGT | ATTCGGCGGTTGACCTCTTT |
| 20 | Endonuclease reverse transcriptase (ERT12) | TRINITY_DN83412_c6_g2_i1 | CGACGTAGCTTCCCAAATGC | GAACCGATTGCGATACACGG |
| 21 | Endonuclease reverse transcriptase (ERT2) | TRINITY_DN83412_c6_g5_i1 | TTGGACGGACATTGTAGCGG | ACTCGGTTGAAATCTGGTCTGT |
